# Supplementary material for: Responses of unicellular predators to cope with the phototoxicity of photosynthetic prey
Source: Nat Commun. 2019 Dec 6;10:5606. doi: 10.1038/s41467-019-13568-6 (PMC6898599; doi:10.1038/s41467-019-13568-6)
Supplement: Supplementary file 1 — Supplementary Information [file 41467_2019_13568_MOESM1_ESM.pdf]

Supplementary Information

Responses of unicellular predators to cope with the phototoxicity of photosynthetic prey

Uzuka, Kobayashi, Onuma et al.

Table of contents:

Supplementary Fig. 1. The changes in the cell number of green or pale S. *elongatus* under dark, low-light, or high-light conditions. 2
Supplementary Fig. 2. The changes in the cell number of *Naegleria* sp. feeding on green or pale S. elongatus under dark, low-light, or high-light conditions. 3
Supplementary Fig. 3. Schematic diagram of the culture conditions for transcriptome analyses. 4
Supplementary Fig. 4. Comparison of the effect of the photosynthetic trait of bacterial prey on mRNA levels of selected genes of *Naegleria* sp., *Acanthamoeba* sp., and *Vannella* sp. 5
Supplementary Fig. 5. Effect of ROS and chlorophyll on mRNA levels of selected *Naegleria* sp. genes 19
Supplementary Fig. 6. Quantitative RT-PCR analyses comparing mRNA levels of respective genes in *Naegleria* sp. in respective culture conditions. 28
Supplementary Table 1. BLAST top hits of 18S rDNA sequences of the three species of amoeboid organisms isolated and used in this study. 30
Supplementary Table 2. Numbers of HiSeq reads that were assigned to mRNA contigs of respective species under respective culture conditions. 32
Supplementary Table 3. GO terms enriched in upregulated genes (FDR < 0.01; edgeR; three biological replicates) when *Naegleria* sp. cultured with green *S. elongatus* prey was transferred from dark to light conditions ($p < 0.05$; GOseq) 33
Supplementary Table 4. GO terms enriched in downregulated genes (FDR < 0.01; edgeR; three biological replicates) when *Naegleria* sp. cultured with green *S. elongatus* prey was transferred from dark to light conditions ($p < 0.05$; GOseq) 35
Supplementary Table 5. GO terms enriched in upregulated genes (FDR < 0.01; edgeR; three biological replicates) when *Acanthamoeba* sp. cultured with green *S. elongatus* prey was transferred from dark to light conditions ($p < 0.05$; GOseq) 37
Supplementary Table 6. GO terms enriched in downregulated genes (FDR < 0.01; edgeR; three biological replicates) when *Acanthamoeba* sp. cultured with green *S. elongatus* prey was transferred from dark to light conditions ($p < 0.05$; GOseq) 39
Supplementary Table 7. Primers used for quantitative RT-PCR in this study (Fig. 4b and Supplementary Fig. 6) 41

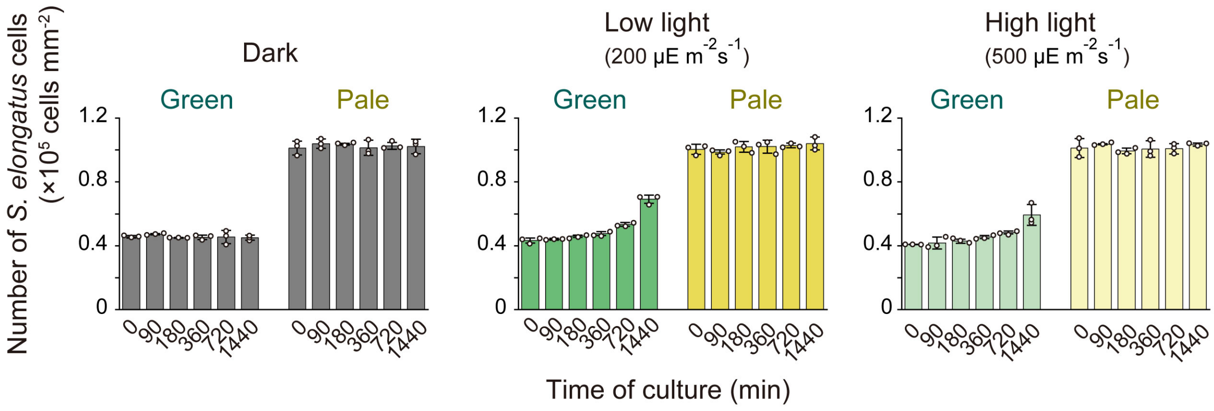

Supplementary Fig. 1. The changes in the cell number of green or pale *S. elongatus* under dark, low-light, or high-light conditions. Green or pale *S. elongatus* without *Naegleria* sp. was cultured in inorganic medium on Petri dishes as in Fig. 2a for 24 h and the change in cell number was determined. The error bar represents the standard deviation of three independent cultures (cultured at the same time). The details are described in Fig. 2. Source data are provided as a Source Data file.

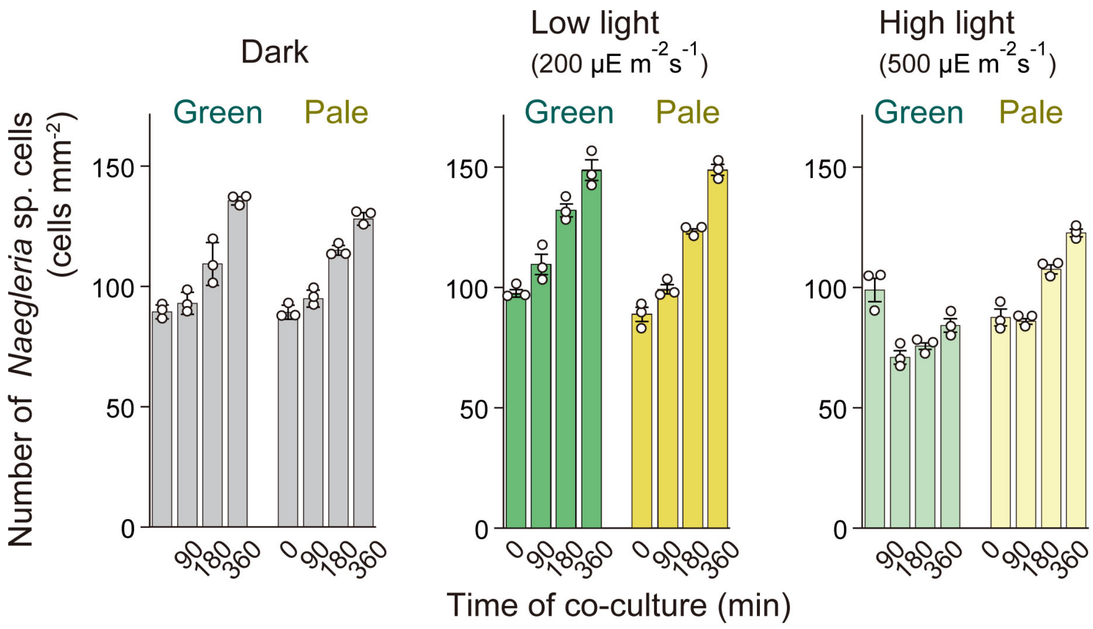

Supplementary Fig. 2. The changes in the cell number of *Naegleria* sp. feeding on green or pale *S. elongatus* under dark, low-light, or high-light conditions. The details are described in Fig. 2. The error bar represents the standard deviation of three independent cultures (cultured at the same time). Source data are provided as a Source Data file.

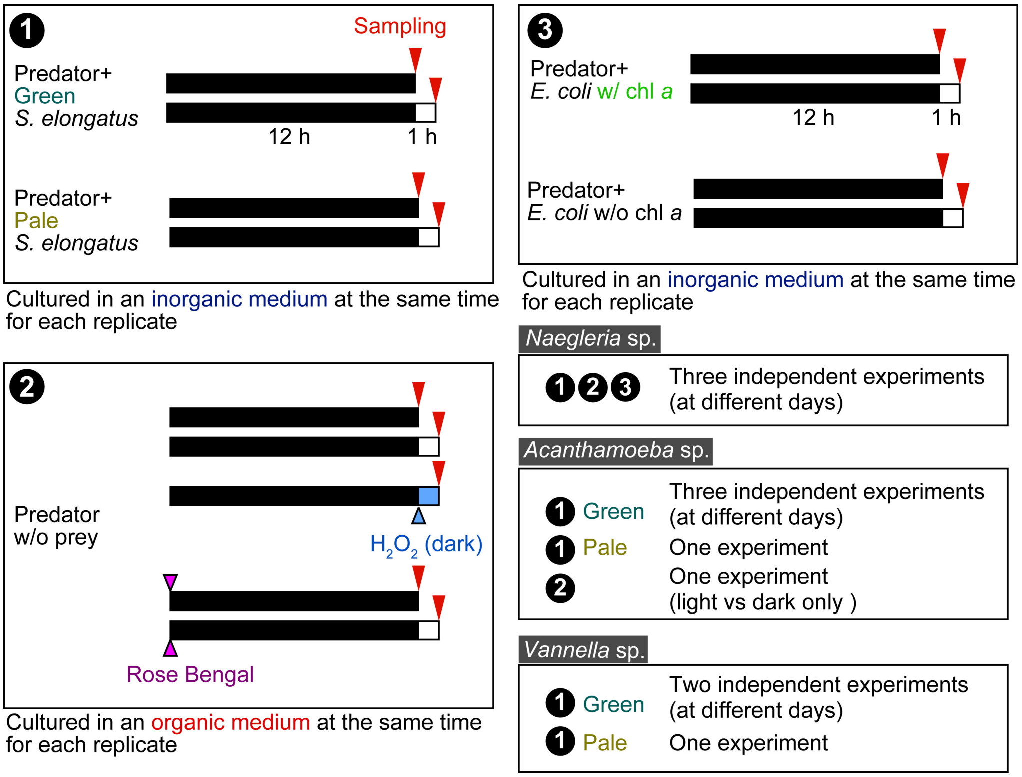

Supplementary Fig. 3. Schematic diagram of the culture conditions for transcriptome analyses. The excavate *Naegleria* sp. and the amoebozoans *Acanthamoeba* sp. and *Vannella* sp. were used as predators. Four types of bacteria, green and pale *S. elongatus* and *E. coli* with or without chlorophyll *a* staining, were used as prey. The co-culture in inorganic medium was incubated at 20 °C in the dark for 12 h and then illuminated (200 µE m^{-2} s^{-1}) for 1 h (group 1 for the three species and group 3 for *Naegleria* sp. only).

Cells were also cultured without bacterial prey in organic medium (group 2; only for *Naegleria* sp. and *Acanthamoeba* sp. because *Vannella* sp. did not grow under this condition). The cells were cultured in the dark for 12 h and were either illuminated (200 µE m^{-2} s^{-1}) or incubated in the dark with 0.1 mM H_2O_2 (*Naegleria* sp. only) for 1 h. For the RB treatment (*Naegleria* sp. only), RB was added to the culture at the onset of the incubation for 12 h in the dark, followed by illumination for 1 h.

As shown in the figure, cultures of each replicate of group 1 (co-culture of the cells and green or pale *S. elongatus* prey), group 2 (culture of the cells without bacterial prey), and group 3 (co-culture of the cells and *E. coli* with or without chlorophyll *a* staining) were performed at the same time for RNA extraction.

Cultures of *Naegleria sp.* in all of the conditions described above were performed three times on different days with *Naegleria* sp. cells newly prepared from the original frozen stock and bacterial prey prepared at the time of use. Because *Acanthamoeba* sp. and *Vannella* sp. growth became unstable after long-term storage, the number of experimental replicates became limited. Cultures of *Acanthamoeba* sp. with green *S. elongatus* prey were performed three times, while those of *Vannella* sp. were performed two times on different days with cells newly prepared from the original frozen stock and bacterial prey prepared at the time of use. Cultures in other conditions were performed once.

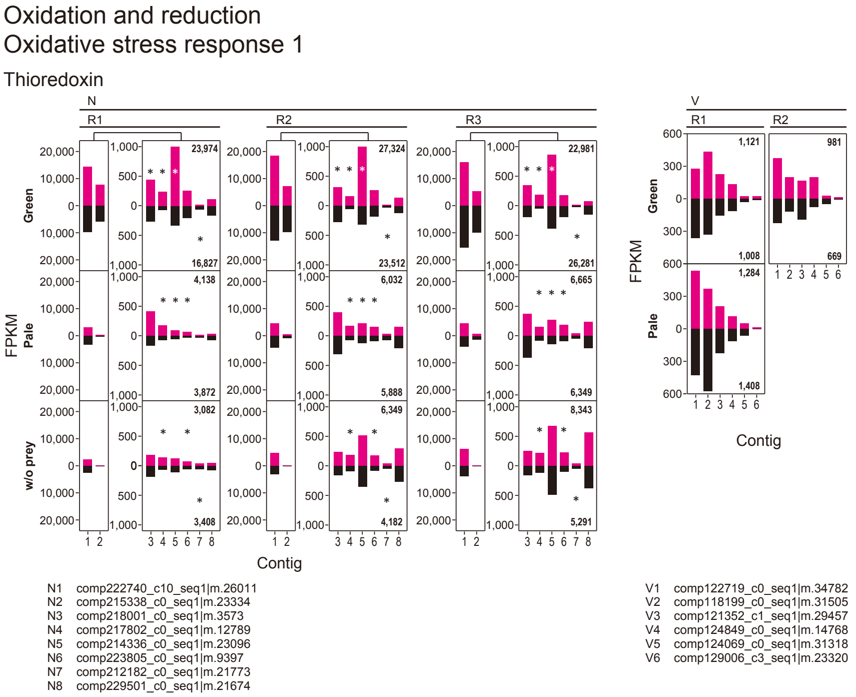

Supplementary Fig. 4. Comparison of the effect of the photosynthetic trait of bacterial prey on mRNA levels of selected genes of *Naegleria* sp., *Acanthamoeba* sp., and *Vannella* sp. Respective cells were co-cultured with green or pale *S. elongatus* prey in the dark for 12 h and then transferred to light (200 μE m^{-2} s^{-1}) conditions for 1h. The cultures with green prey were performed three times (R1, R2, and R3) for *Naegleria* sp. and *Acanthamoeba* sp., while those for *Vannella* sp. were performed two times (R1 and R2) independently on different days. To examine the effect of illumination that is independent of prey, respective species were cultured in organic growth medium without bacterial prey in the dark for 12 h and then transferred to light conditions for 1 h. *Naegleria* sp. cultures were also performed three times independently on different days (R1, R2, and R3), while cultures of *Acanthamoeba* sp. and *Vannella* sp. were performed once. Graphs show the mRNA levels (FPKM values) of contigs (genes) that are related to oxidation and reduction/oxidative stress responses, carotenoid synthesis, DNA repair, respiration and oxygen-consuming metabolism or encoding myosin, actin and PAO-like proteins under dark (black bar) and light (magenta bar) conditions. Each bar corresponds to one contig. For example, 8, 11, and 6 contigs (genes) encoded thioredoxin in *Naegleria sp.*, *Acanthamoeba* sp., and *Vannella* sp., respectively. Numbers in the graph indicate total FPKM values in respective conditions. The contig IDs are shown beside each graph. *, FDR < 0.01 (edgeR) only when datasets of three independent replicates were obtained. The details of the culture conditions are described in Supplementary Fig. 3.

Oxidation and reduction
Oxidative stress response 1

Thioredoxin

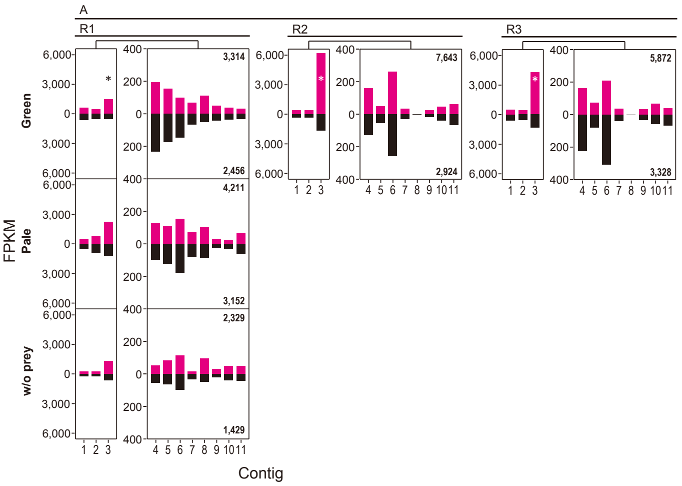

A1 comp191596_c1_seq2|m.42213
A2 comp191030_c0_seq1|m.46136
A3 comp188618_c0_seq1|m.31761
A4 comp197048_c0_seq1|m.7755
A5 comp192368_c0_seq1|m.33075
A6 comp194676_c0_seq1|m.19143
A7 comp190977_c1_seq1|m.39093
A8 comp191774_c0_seq1|m.37757
A9 comp195144_c0_seq1|m.32433
A10 comp193291_c1_seq4|m.35492
A11 comp193004_c0_seq1|m.19663

Supplementary Fig. 4. Comparison of the effect of the photosynthetic trait of bacterial prey on mRNA levels of selected genes of *Naegleria* sp., *Acanthamoeba* sp., and *Vannella* sp. (continued)

Oxidation and reduction
Oxidative stress response 2

Glutathione synthetase

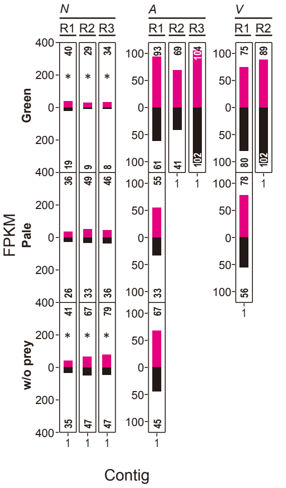

N1 comp170906_c0_seq1|m.12558

A1 comp198022_c1_seq5|m.13728

V1 comp126782_c0_seq1|m.13264

Carbonyl reductase

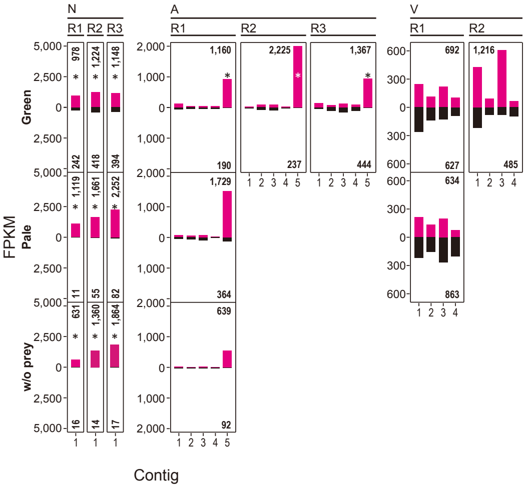

N1 comp222757_c0_seq1|m.17440

A1 comp193008_c0_seq1|m.24805
A2 comp195979_c0_seq1|m.27346
A3 comp192631_c0_seq2|m.37586
A4 comp190819_c0_seq1|m.38965
A5 comp193008_c0_seq1|m.24804

V1 comp126764_c0_seq1|m.31102
V2 comp121793_c0_seq1|m.35963
V3 comp122682_c0_seq2|m.16871
V4 comp121793_c1_seq1|m.39085

Glutathion S-transferase

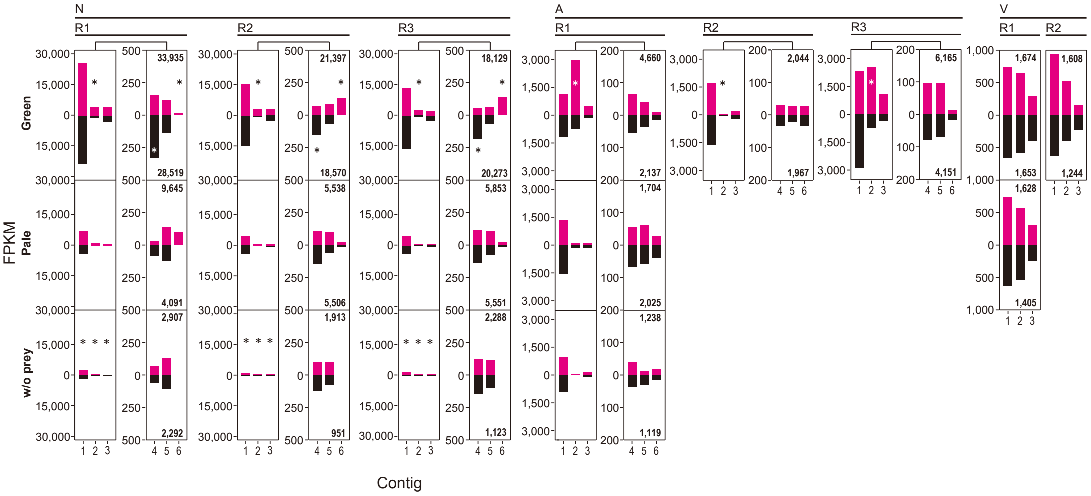

N1 comp222120_c0_seq1|m.19841
N2 comp215852_c0_seq1|m.19804
N3 comp220161_c1_seq1|m.17653
N4 comp219327_c0_seq1|m.18450
N5 comp171992_c0_seq1|m.19016
N6 comp219524_c0_seq1|m.20621

A1 comp188728_c0_seq1|m.30477
A2 comp190111_c0_seq1|m.44542
A3 comp193211_c0_seq1|m.42968
A4 comp191147_c0_seq2|m.40291
A5 comp190522_c0_seq1|m.39396
A6 comp191234_c0_seq2|m.34399

V1 comp98094_c0_seq1|m.26854
V2 comp106525_c0_seq1|m.37532
V3 comp125662_c0_seq1|m.37473

Supplementary Fig. 4. Comparison of the effect of the photosynthetic trait of bacterial prey on mRNA levels of selected genes of *Naegleria* sp., *Acanthamoeba* sp., and *Vannella* sp. (continued)

Oxidation and reduction
Oxidative stress response 3

Glutathione peroxidase

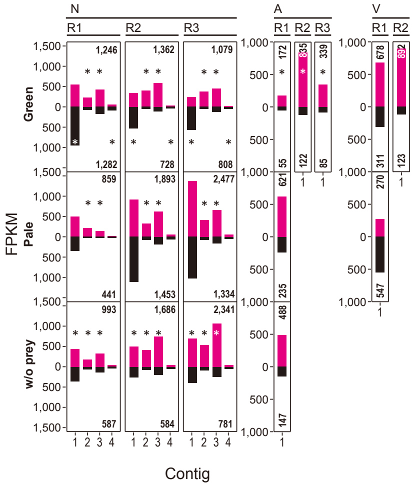

N1 comp197051_c1_seq2|m.22797
N2 comp227059_c0_seq3|m.1915
N3 comp226183_c1_seq1|m.3708
N4 comp230203_c0_seq1|m.20301

A1 comp190730_c0_seq1|m.38776

V1 comp118319_c0_seq2|m.24239

Glutaredoxin

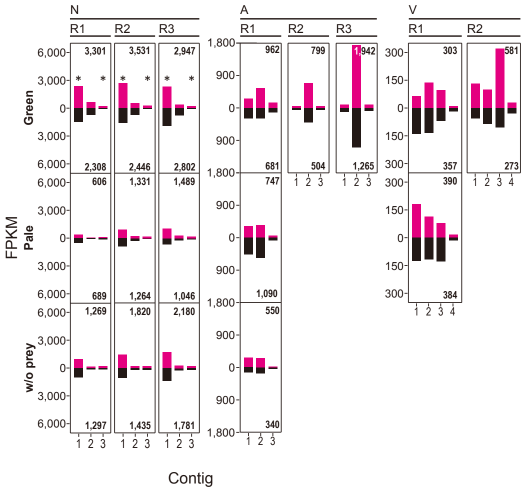

N1 comp197043_c0_seq1|m.26229
N2 comp196883_c0_seq1|m.24409
N3 comp221174_c0_seq1|m.16477

A1 comp199540_c0_seq1|m.12891
A2 comp127545_c0_seq1|m.49958
A3 comp189732_c0_seq1|m.39341

V1 comp117553_c0_seq1|m.45875
V2 comp122551_c0_seq1|m.17789
V3 comp120750_c0_seq1|m.35507
V4 comp129187_c1_seq1|m.1393

Peptide methionine sulfoxide reductase

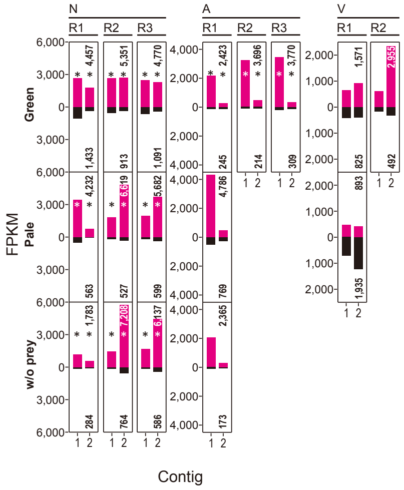

N1 comp161358_c0_seq1|m.4904
N2 comp224705_c0_seq2|m.7781

A1 comp191467_c0_seq1|m.39140
A2 comp196146_c1_seq1|m.42352

V1 comp107284_c0_seq1|m.23595
V2 comp121801_c1_seq1|m.29460

Catalase

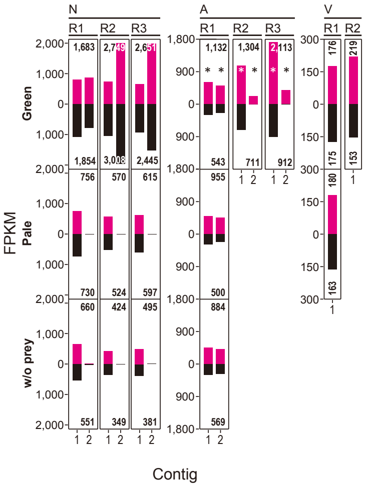

N1 comp192408_c0_seq1|m.4650
N2 comp174950_c0_seq1|m.10133

A1 comp188885_c0_seq1|m.25786
A2 comp188885_c0_seq1|m.25787

V1 comp123359_c0_seq1|m.13841

Supplementary Fig. 4. Comparison of the effect of the photosynthetic trait of bacterial prey on mRNA levels of selected genes of *Naegleria* sp., *Acanthamoeba* sp., and *Vannella* sp. (continued)

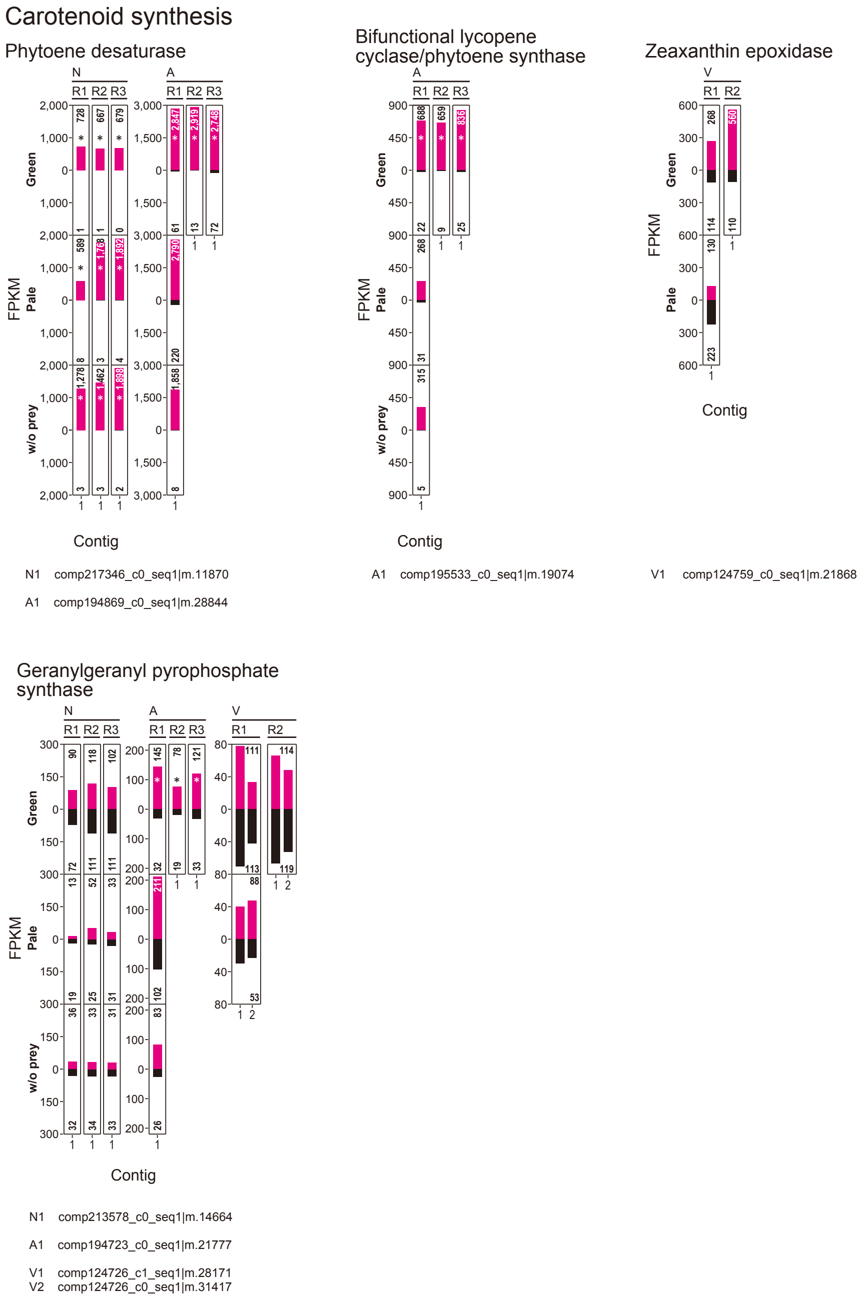

Supplementary Fig. 4. Comparison of the effect of the photosynthetic trait of bacterial prey on mRNA levels of selected genes of *Naegleria* sp., *Acanthamoeba* sp., and *Vannella* sp. (continued)

DNA repair

DNA-(apurinic or apyrimidinic site) lyase

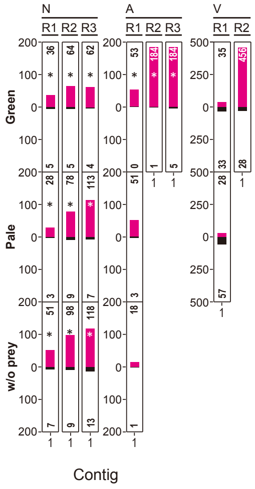

N1 comp225006_c0_seq1|m.7768

A1 comp199388_c0_seq1|m.9331

V1 comp126066_c0_seq2|m.29588

RAD23

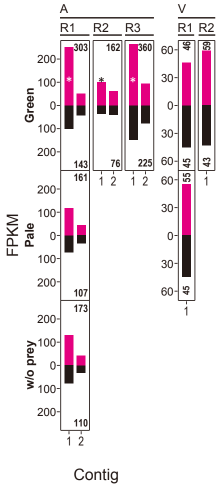

A1 comp191210_c0_seq1|m.31519
A2 comp193102_c0_seq1|m.31176

V1 comp123008_c0_seq1|m.15170

Deoxyribodipyrimidine photo-lyase

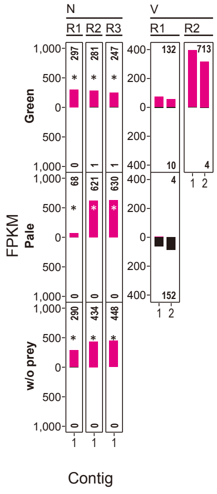

N1 comp222533_c0_seq1|m.15479

V1 comp125035_c0_seq1|m.12561
V2 comp127813_c1_seq1|m.15993

Supplementary Fig. 4. Comparison of the effect of the photosynthetic trait of bacterial prey on mRNA levels of selected genes of *Naegleria* sp., *Acanthamoeba* sp., and *Vannella* sp. (continued)

Respiration 1

COX11

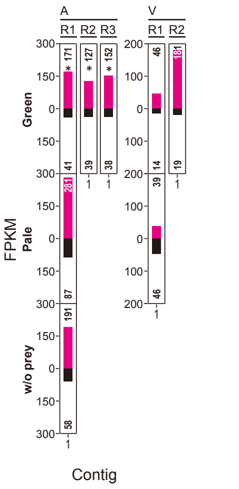

A1 comp193195_c0_seq1|m.37792

V1 comp122488_c0_seq2|m.24697

COX15

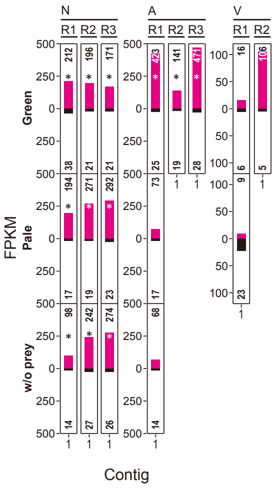

N1 comp228272_c0_seq1|m.12304

A1 comp192893_c0_seq1|m.25498

V1 comp125185_c0_seq2|m.16544

TIM14

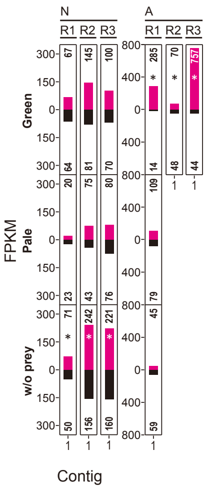

N1 comp220797_c0_seq1|m.5849

A1 comp192514_c0_seq1|m.44559

TIM16

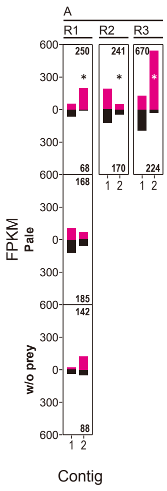

A1 comp181094_c0_seq2|m.48439

A2 comp184602_c0_seq1|m.48738

TIM17

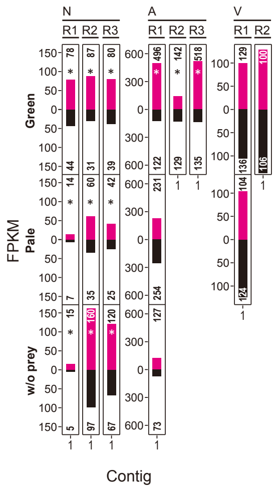

N1 comp186302_c0_seq1|m.19129

A1 comp178054_c1_seq1|m.47330

V1 comp120032_c0_seq1|m.21088

Supplementary Fig. 4. Comparison of the effect of the photosynthetic trait of bacterial prey on mRNA levels of selected genes of *Naegleria* sp., *Acanthamoeba* sp., and *Vannella* sp. (continued)

Respiration 2

TIM23

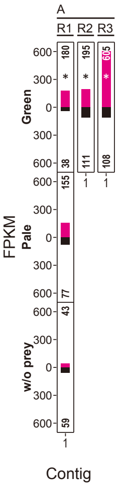

A1 comp190408_c0_seq1|m.49523

TIM44

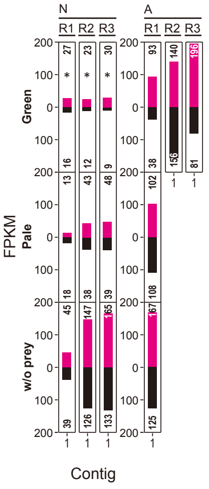

N1 comp182943_c0_seq1|m.12522

A1 comp192049_c0_seq1|m.28999

Prohibitin 1

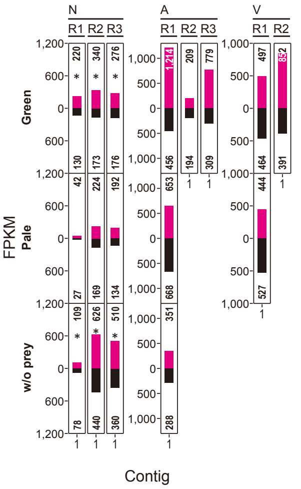

N1 comp228209_c0_seq1|m.19680

A1 comp189959_c0_seq1|m.42468

V1 comp122032_c0_seq1|m.30636

Prohibitin 2

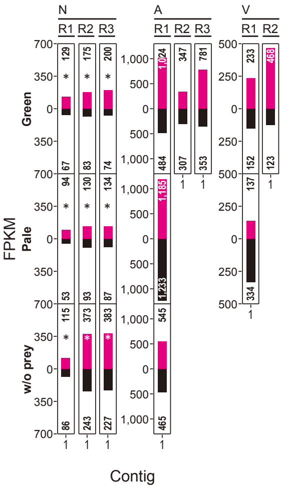

N1 comp227900_c0_seq1|m.15768

A1 comp192167_c0_seq1|m.37994

V1 comp122381_c0_seq1|m.21229

Supplementary Fig. 4. Comparison of the effect of the photosynthetic trait of bacterial prey on mRNA levels of selected genes of *Naegleria* sp., *Acanthamoeba* sp., and *Vannella* sp. (continued)

Oxygen consuming metabolisms

Methylsterol monooxygenase

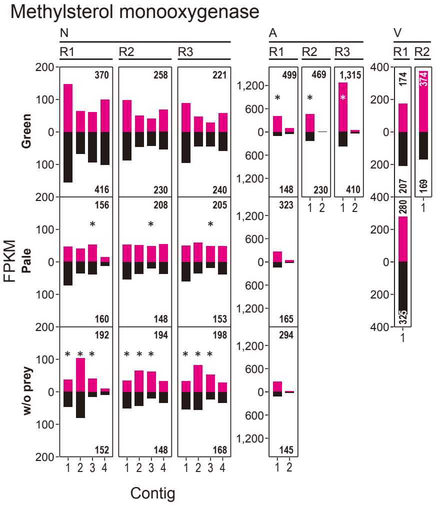

N1 comp228408_c0_seq1|m.17822
N2 comp224022_c0_seq1|m.7534
N3 comp228514_c0_seq1|m.11837
N4 comp218051_c0_seq1|m.11145

A1 comp193498_c0_seq1|m.19446
A2 comp198885_c0_seq1|m.33502

V1 comp121846_c0_seq1|m.24109

Squalene monooxygenase

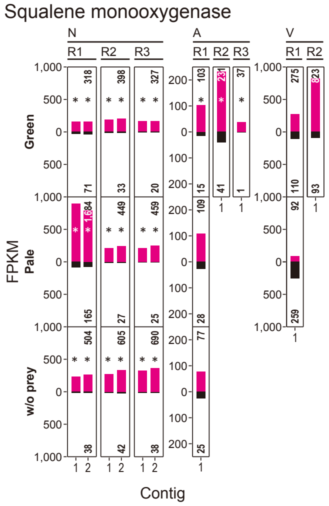

N1 comp216080_c1_seq1|m.5734
N2 comp216080_c0_seq1|m.19550

A1 comp195623_c0_seq1|m.11809

V1 comp122050_c0_seq1|m.9848

L-gulonolactone oxidase

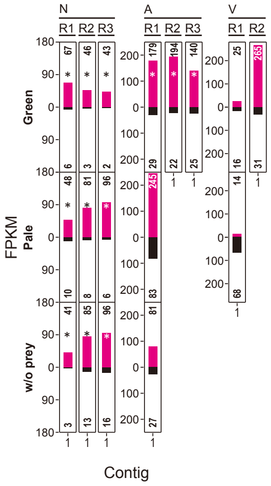

N1 comp220489_c0_seq1|m.13475

A1 comp194339_c0_seq2|m.27513

V1 comp128213_c0_seq2|m.16856

Supplementary Fig. 4. Comparison of the effect of the photosynthetic trait of bacterial prey on mRNA levels of selected genes of *Naegleria* sp., *Acanthamoeba* sp., and *Vannella* sp. (continued)

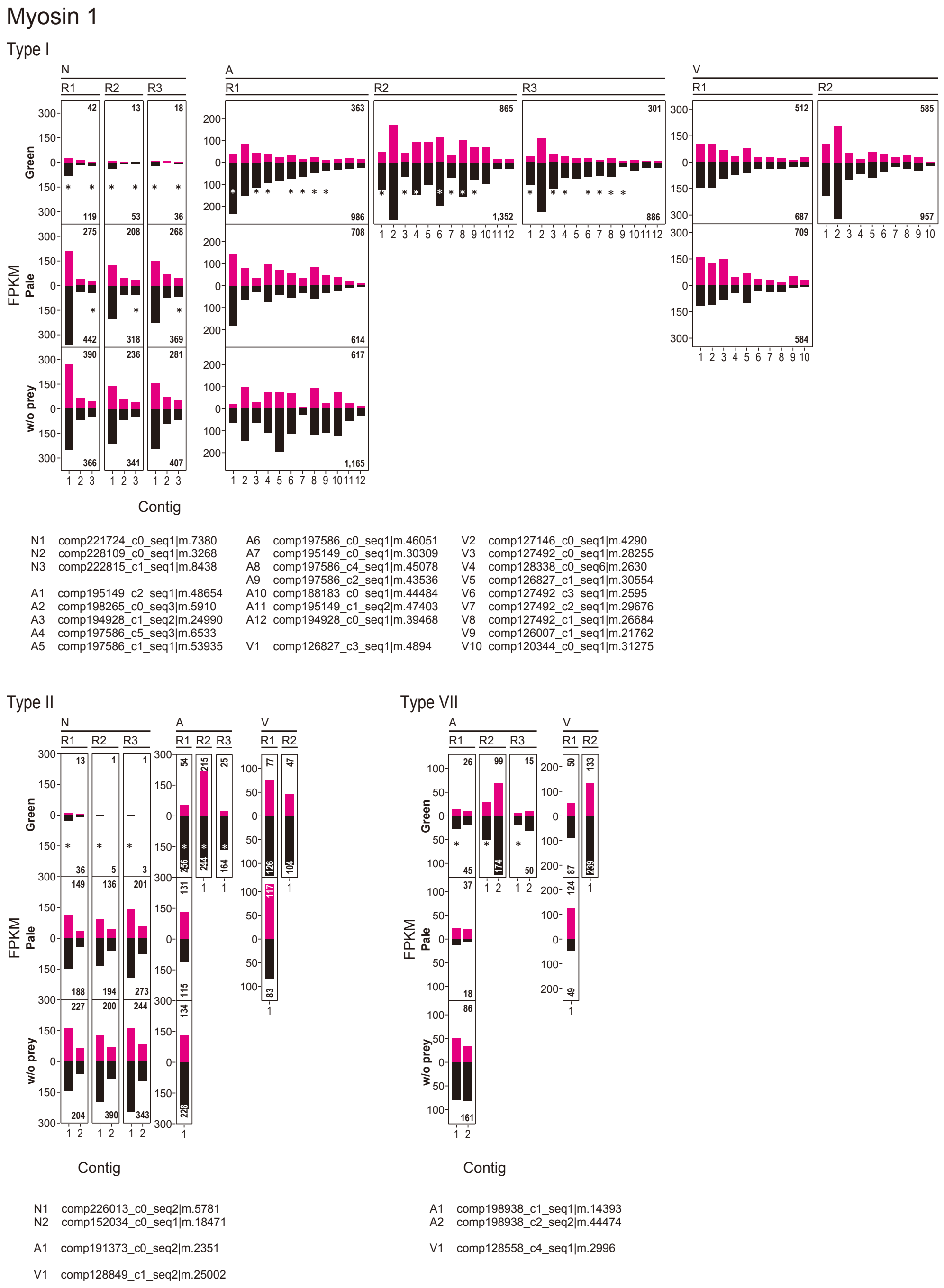

Supplementary Fig. 4. Comparison of the effect of the photosynthetic trait of bacterial prey on mRNA levels of selected genes of *Naegleria* sp., *Acanthamoeba* sp., and *Vannella* sp. (continued)

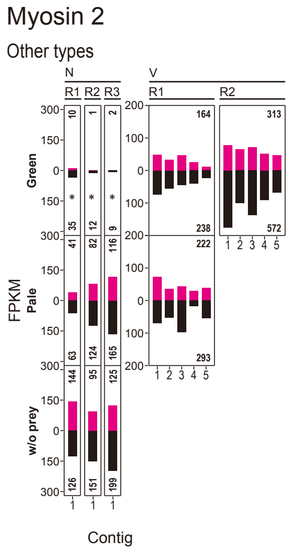

N1 comp224888_c0_seq1|m.3961

V1 comp128849_c2_seq1|m.627
V2 comp126007_c2_seq1|m.27590
V3 comp128558_c3_seq1|m.25085
V4 comp126007_c0_seq1|m.22111
V5 comp126007_c4_seq1|m.28061

Supplementary Fig. 4. Comparison of the effect of the photosynthetic trait of bacterial prey on mRNA levels of selected genes of *Naegleria* sp., *Acanthamoeba* sp., and *Vannella* sp. (continued)

Actin

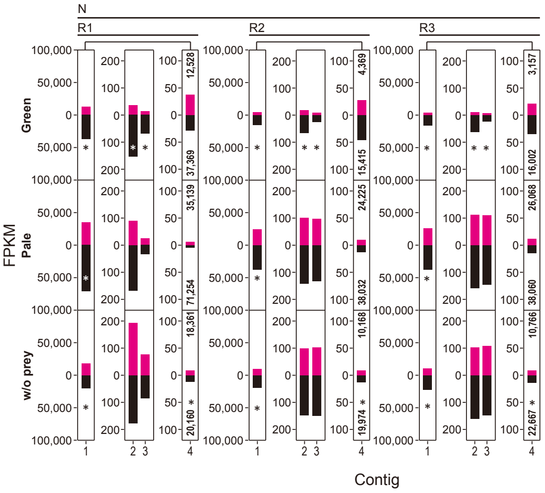

N1 comp226268_c0_seq1|m.15372
N2 comp218105_c0_seq1|m.17135
N3 comp212496_c0_seq1|m.10790
N4 comp225048_c0_seq1|m.5103

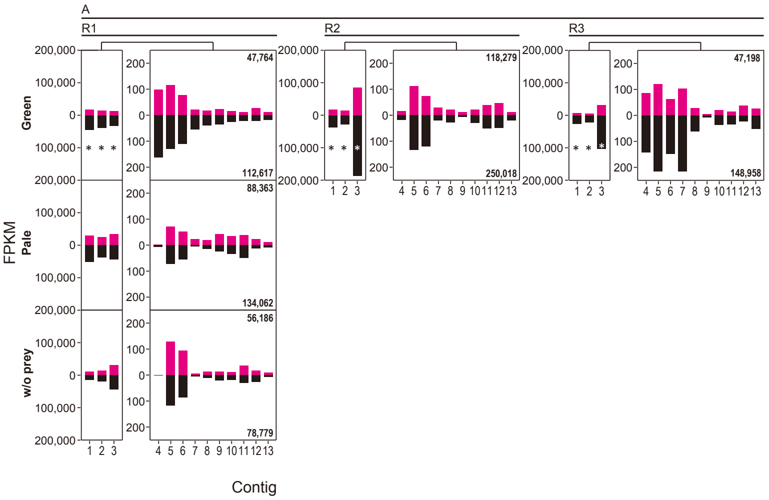

A1 comp197233_c0_seq1|m.48321
A2 comp197233_c3_seq1|m.51575
A3 comp197233_c1_seq1|m.54766
A4 comp192995_c0_seq1|m.38899
A5 comp193353_c0_seq1|m.5798
A6 comp189645_c0_seq1|m.55912
A7 comp191378_c0_seq3|m.30974
A8 comp197190_c1_seq1|m.24458
A9 comp195956_c0_seq1|m.9932
A10 comp198783_c0_seq1|m.13041
A11 comp197962_c1_seq1|m.12530
A12 comp198043_c0_seq5|m.9355
A13 comp195269_c1_seq1|m.31367

Supplementary Fig. 4. Comparison of the effect of the photosynthetic trait of bacterial prey on mRNA levels of selected genes of *Naegleria* sp., *Acanthamoeba* sp., and *Vannella* sp. (continued)

Actin

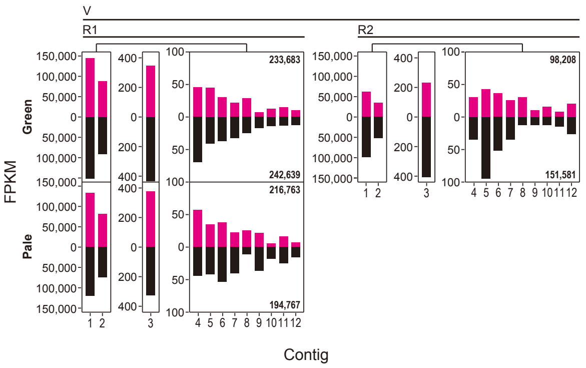

V1 comp127126_c1_seq1|m.30423
V2 comp127126_c3_seq1|m.35979
V3 comp121056_c0_seq1|m.15382
V4 comp126422_c0_seq1|m.18167
V5 comp126520_c0_seq1|m.22802
V6 comp124568_c0_seq2|m.11270
V7 comp126591_c0_seq1|m.22524
V8 comp125992_c0_seq2|m.31100
V9 comp123034_c0_seq2|m.25940
V10 comp128124_c0_seq5|m.17121
V11 comp124299_c0_seq1|m.15318
V12 comp126794_c0_seq2|m.26626

Supplementary Fig. 4. Comparison of the effect of the photosynthetic trait of bacterial prey on mRNA levels of selected genes of *Naegleria* sp., *Acanthamoeba* sp., and *Vannella* sp. (continued)

PAO-like protein

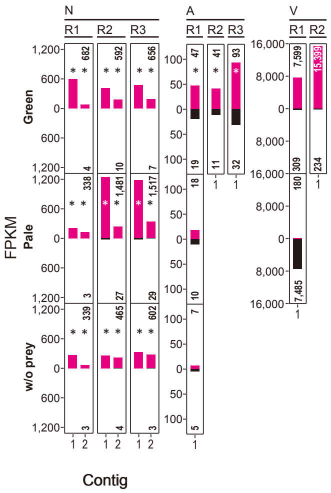

N1 comp196469_c0_seq1|m.12866
N2 comp224603_c0_seq1|m.12628

A1 comp195620_c0_seq3|m.26633

V1 comp127201_c0_seq1|m.25614

Supplementary Fig. 4. Comparison of the effect of the photosynthetic trait of bacterial prey on mRNA levels of selected genes of *Naegleria* sp., *Acanthamoeba* sp., and *Vannella* sp. (continued)

Oxidation and reduction
Oxidative stress response 1
Thioredoxin

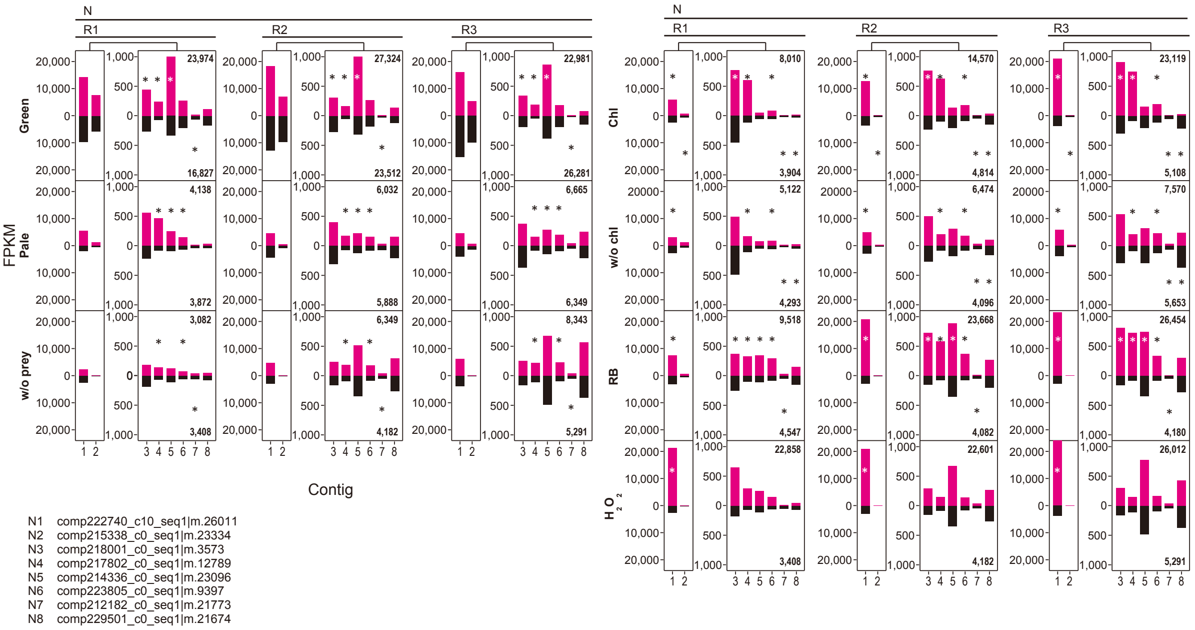

Supplementary Fig. 5. Effect of ROS and chlorophyll on mRNA levels of selected *Naegleria* sp. genes. To examine the effect of chlorophyll *a*, *Naegleria* sp. was cultured with *E. coli* stained with/without chlorophyll *a* in the dark in inorganic medium for 12 h and then transferred to light (200 μE m^{-2} s^{-1}) conditions for 1 h. To examine the effect of singlet oxygen, *Naegleria* sp. was cultured without bacterial prey in organic medium with 30 nM RB, which produces singlet oxygen by illumination, in the dark for 12 h and then transferred to light conditions for 1 h. To examine the effects of H_2O_2, *Naegleria* sp. was cultured without bacterial prey in organic medium for 12 h in the dark and treated with 0.1 mM H_2O_2 in the dark for 1 h. Cultures under respective conditions were performed three times independently on different days (R1, R2, and R3). Graphs show the levels of mRNA (FPKM values) that are related to oxidation and reduction/oxidative stress responses, carotenoid synthesis, DNA repair, respiration and oxygen-consuming metabolism or encoding myosin, actin and PAO-like proteins under dark (black bar) and light (magenta bar) conditions, except for H_2O_2. In the case of H_2O_2 treatment, the black bar indicates the value just before the addition of H_2O_2 and the magenta bar indicates the value 1 h after its addition in the dark. Each bar corresponds to one contig (gene). For example, four contigs (genes) encoded glutathione peroxidase in *Naegleria* sp. Numbers in the graph indicate the total FPKM values in respective conditions. The contig IDs are shown below each graph. For comparison, the values in the cultures with green or pale *S. elongatus* prey and illumination alone (w/o prey) (shown in Supplementary Fig. 4) are redisplayed. *, FDR < 0.01 (edgeR; three biological replicates). The details of the culture conditions are described in Supplementary Fig. 3.

Oxidation and reduction
Oxidative stress response 2

Glutathione synthetase

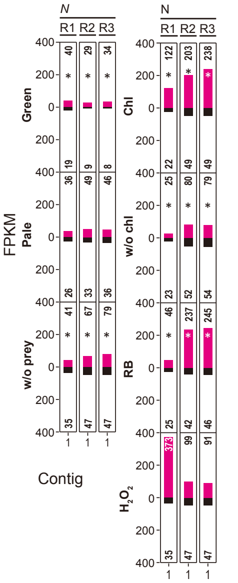

N1 comp170906_c0_seq1|m.12558

Carbonyl reductase

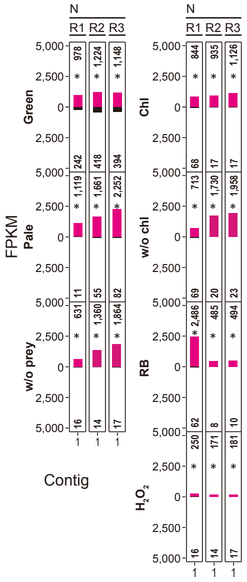

N1 comp222757_c0_seq1|m.17440

Glutathione S-transferase

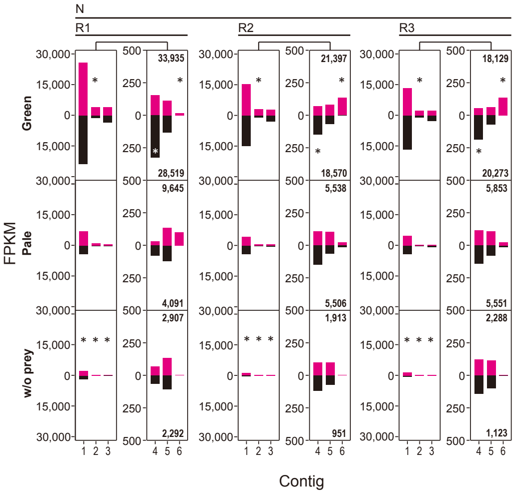

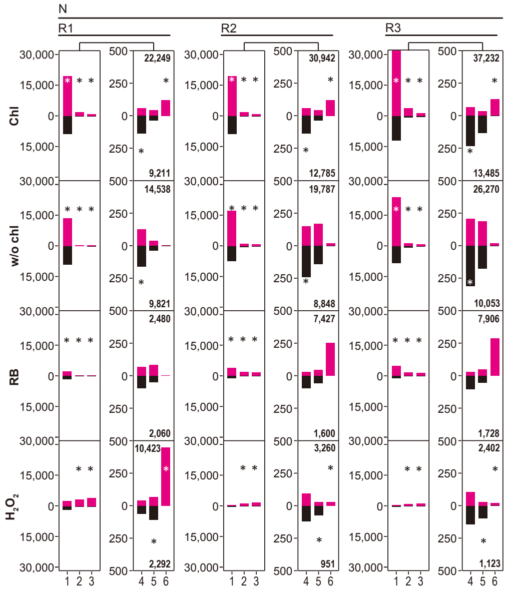

N1 comp222120_c0_seq1|m.19841
N2 comp215852_c0_seq1|m.19804
N3 comp220161_c1_seq1|m.17653
N4 comp219327_c0_seq1|m.18450
N5 comp171992_c0_seq1|m.19016
N6 comp219524_c0_seq1|m.20621

Supplementary Fig. 5. Effect of ROS and chlorophyll on mRNA levels of selected *Naegleria* sp. genes. (continued)

Oxidation and reduction
Oxidative stress response 3

Glutathione peroxidase

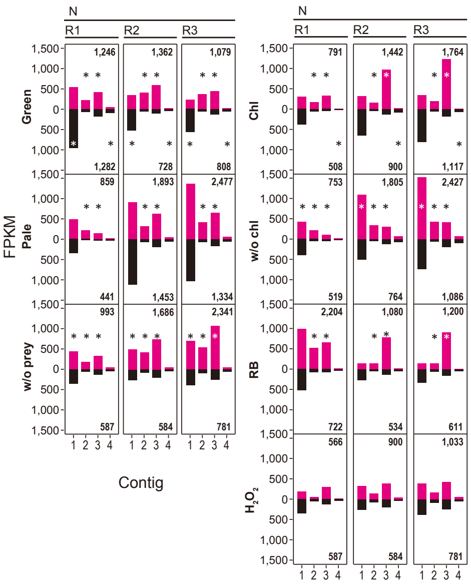

N1 comp197051_c1_seq2|m.22797
N2 comp227059_c0_seq3|m.1915
N3 comp226183_c1_seq1|m.3708
N4 comp230203_c0_seq1|m.20301

Glutaredoxin

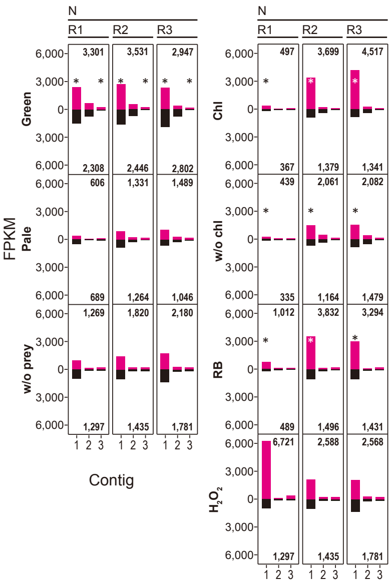

N1 comp197043_c0_seq1|m.26229
N2 comp196883_c0_seq1|m.24409
N3 comp221174_c0_seq1|m.16477

Peptide methionine sulfoxide reductase

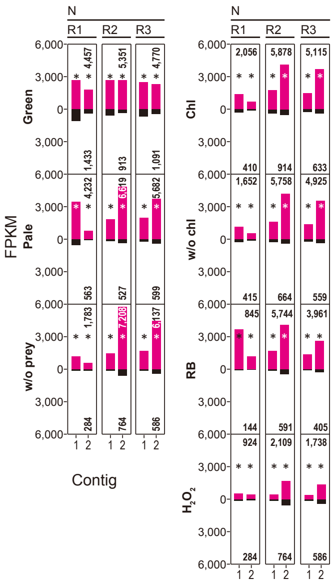

N1 comp161358_c0_seq1|m.4904
N2 comp224705_c0_seq2|m.7781

Supplementary Fig. 5. Effect of ROS and chlorophyll on mRNA levels of selected *Naegleria* sp. genes. (continued)

Carotenoid synthesis

Phytoene desaturase

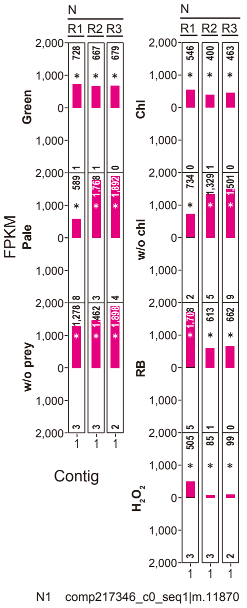

DNA repair

Deoxyribodipyrimidine photo-lyase

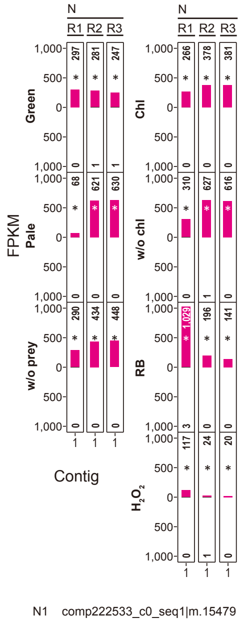

DNA-(apurinic or apyrimidinic site) lyase

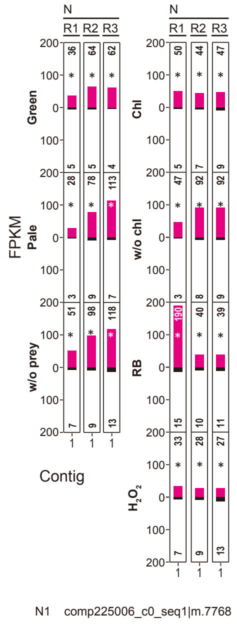

Supplementary Fig. 5. Effect of ROS and chlorophyll on mRNA levels of selected *Naegleria* sp. genes. (continued)

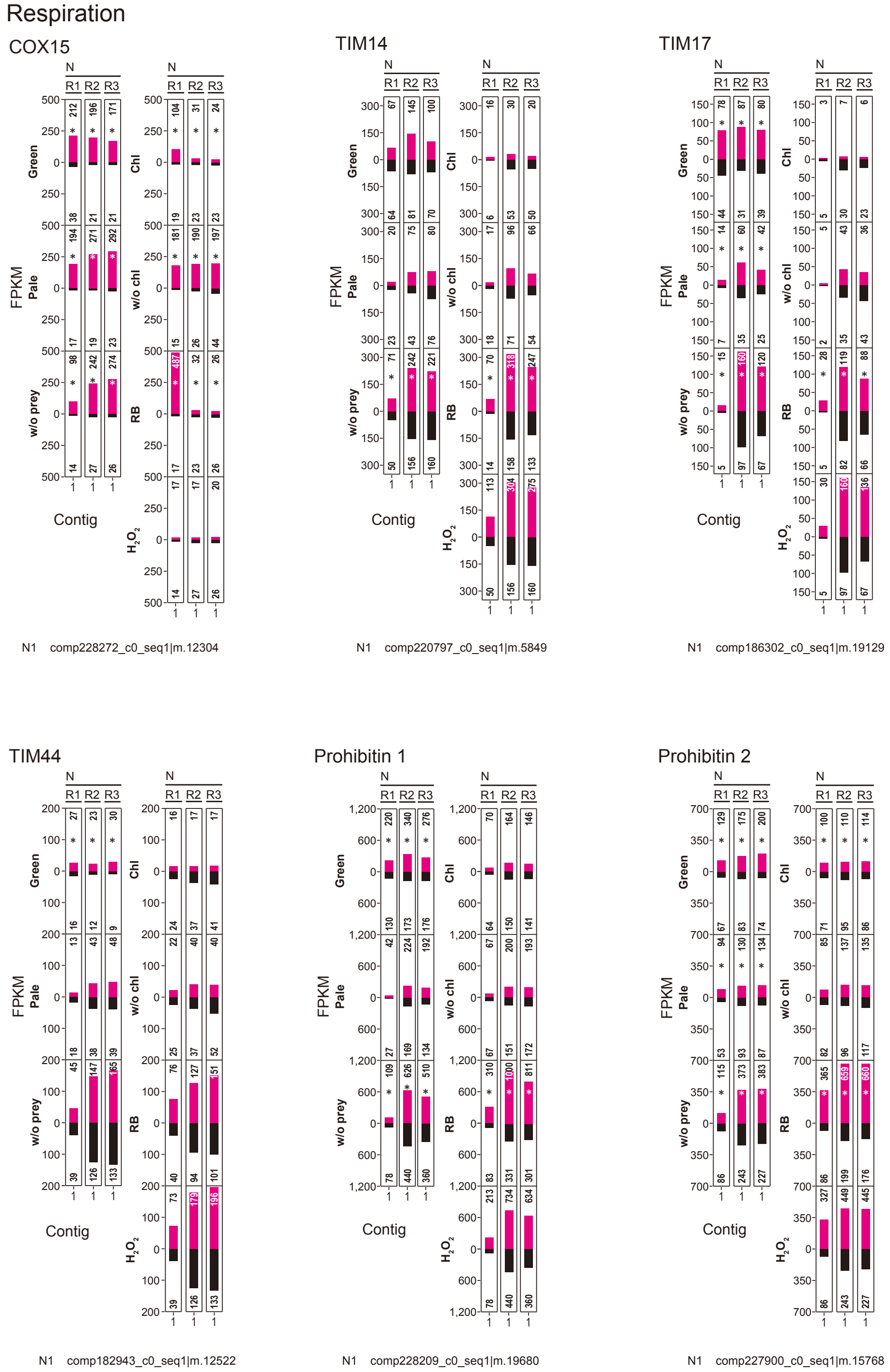

Supplementary Fig. 5. Effect of ROS and chlorophyll on mRNA levels of selected *Naegleria* sp. genes. (continued)

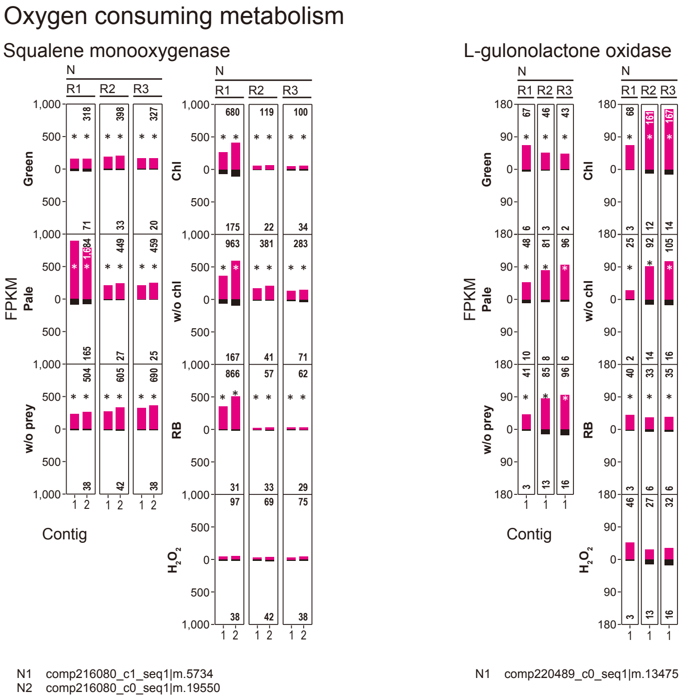

Supplementary Fig. 5. Effect of ROS and chlorophyll on mRNA levels of selected *Naegleria* sp. genes. (continued)

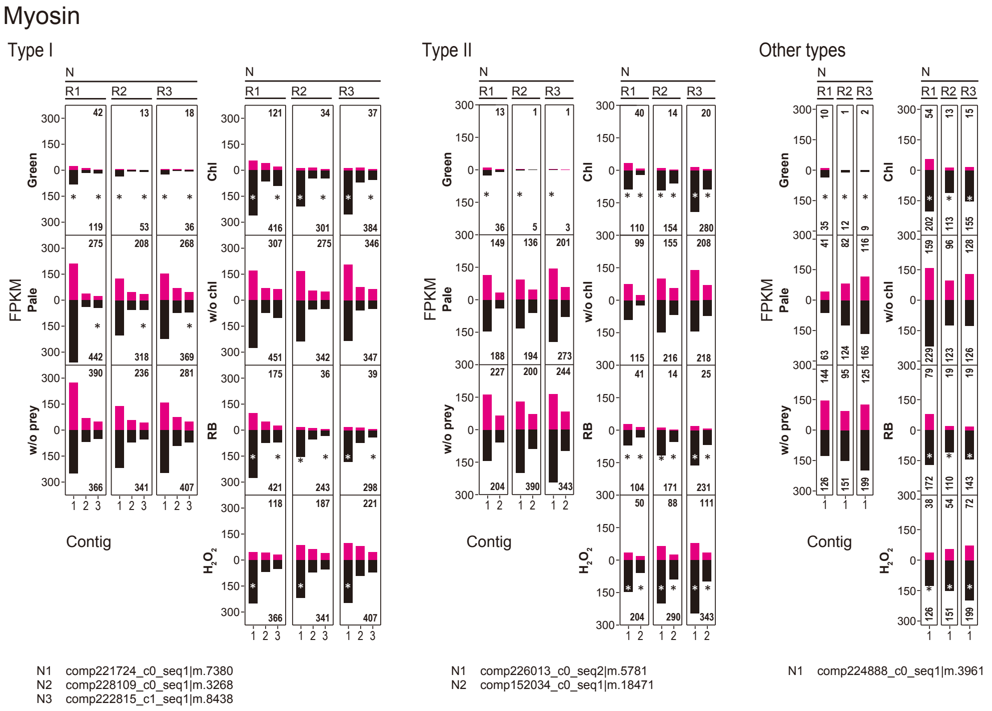

Supplementary Fig. 5. Effect of ROS and chlorophyll on mRNA levels of selected *Naegleria* sp. genes. (continued)

Actin

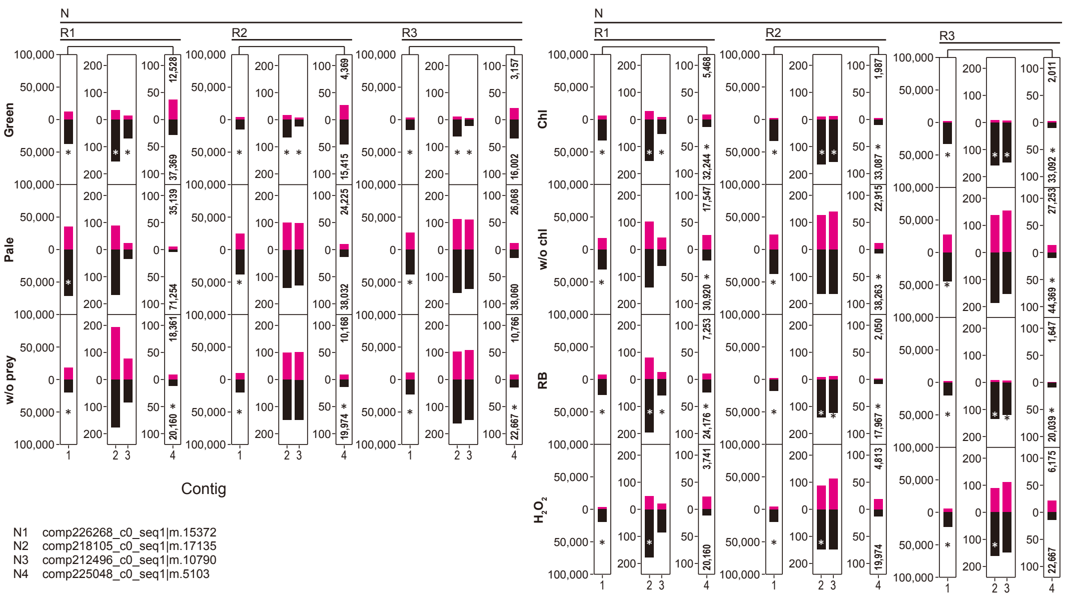

Supplementary Fig. 5. Effect of ROS and chlorophyll on mRNA levels of selected *Naegleria* sp. genes. (continued)

PAO-like protein

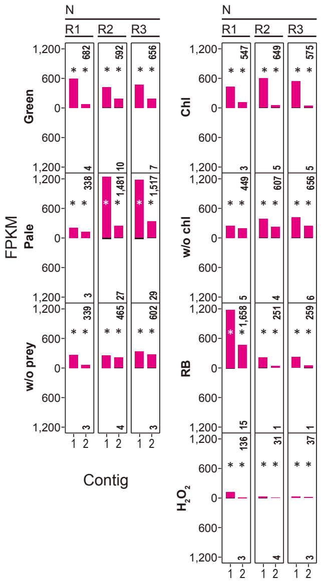

N1 comp196469_c0_seq1|m.12866
N2 comp224603_c0_seq1|m.12628

Supplementary Fig. 5. Effect of ROS and chlorophyll on mRNA levels of selected *Naegleria* sp. genes. (continued)

Naegleria sp. qRT-PCR

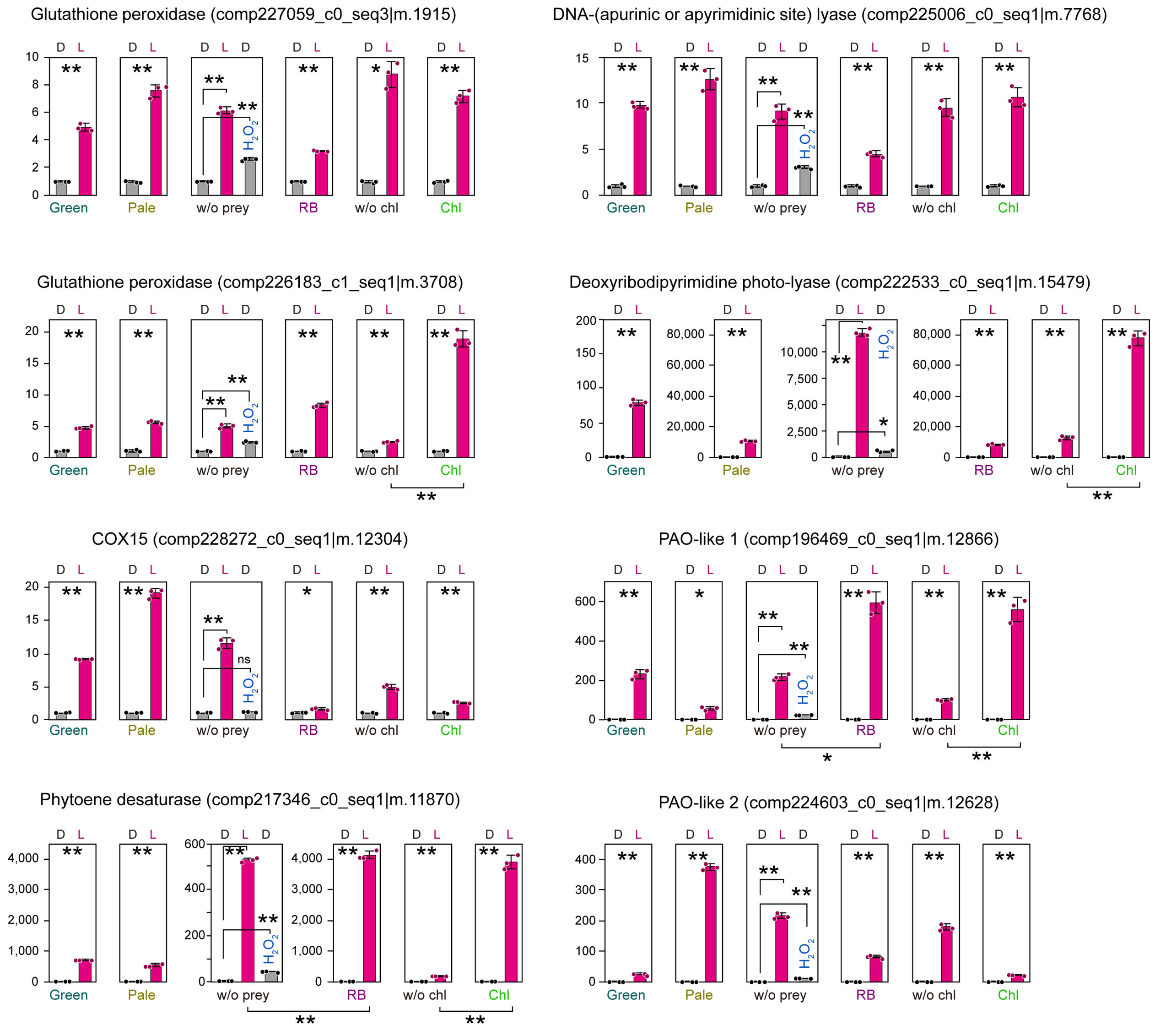

Supplementary Fig. 6. Quantitative RT-PCR analyses comparing mRNA levels of respective genes in *Naegleria* sp. in respective culture conditions. The corresponding RNA-seq results are shown in Fig. 4b and Supplementary Fig. 5. The details of the culture conditions are described in Supplementary Fig. 3. *EF1α* (comp225284_c0_seq1|m.13820) was used as an internal control. The level in the dark conditions (or before H_2O_2 addition) was defined as 1.0. The error bar represents the standard deviation of three replicates (three sets of RNA samples prepared from three sets of *Naegleria* sp. cultures that were performed at the same time). *$p < 0.05$; **$p < 0.005$; ns, not statistically significant (*t*-test). Source data are provided as a Source Data file.

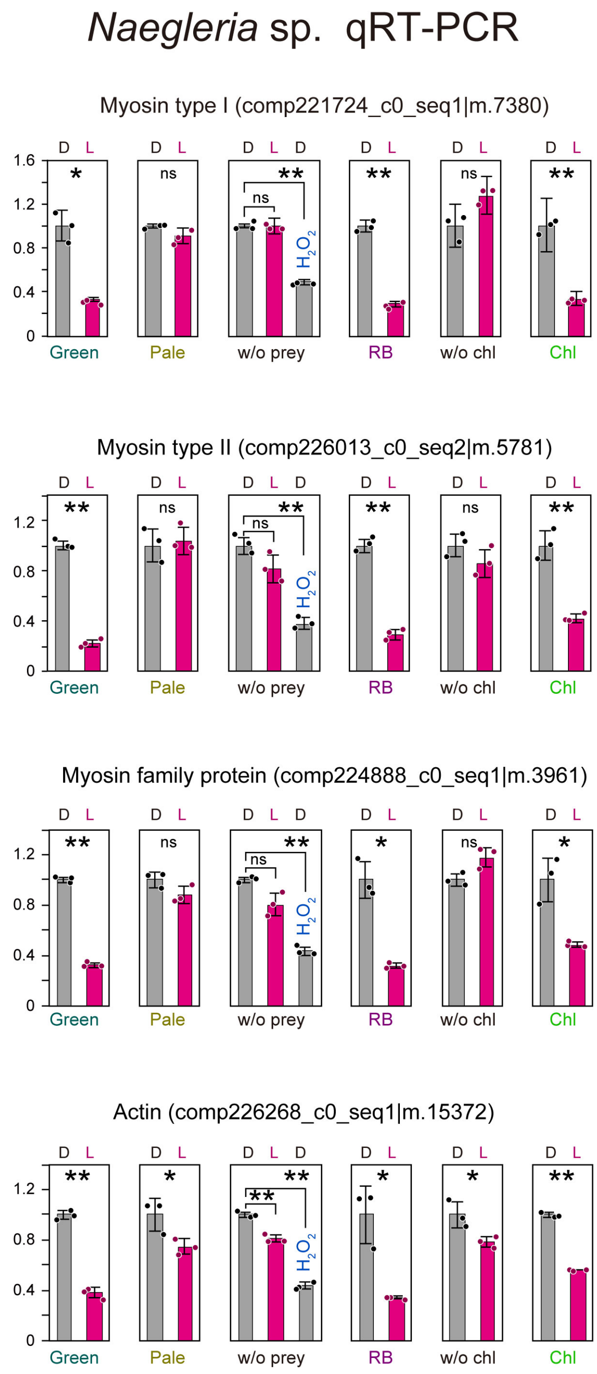

Supplementary Fig. 6. Quantitative RT-PCR analyses comparing mRNA levels of respective genes in *Naegleria* sp. in respective culture conditions. (continued)

Supplementary Table 1. BLAST top hits of 18S rDNA sequences of the three species of amoeboid organisms isolated and used in this study.

Species	Accession number	Classification	E-value
Naegleria sp. KDN1 (query; this study)	LC368140		
**Top 10 hits**			
Naegleria jejuensis JJ42108A	MF113400.1	Heterolobosea; Schizopyrenida	0
Naegleria pagei A22/I	DQ768714.1	Heterolobosea; Schizopyrenida	0
Naegleria pagei 4830/I	DQ768721.1	Heterolobosea; Schizopyrenida	0
Naegleria gruberi EGB	MG699123.1	Heterolobosea; Schizopyrenida	0
Naegleria gruberi NEG-M	AB298288.1	Heterolobosea; Schizopyrenida	0
Naegleria gruberi	M18732.1	Heterolobosea; Schizopyrenida	0
Naegleria sp. 62K4	JQ271695.1	Heterolobosea; Schizopyrenida	0
Naegleria sp. COHH 64	GU320598.1	Heterolobosea; Schizopyrenida	0
Naegleria australiensis BCHV5	JQ271699.1	Heterolobosea; Schizopyrenida	0
Naegleria fultoni 4796/I	DQ768719.1	Heterolobosea; Schizopyrenida	0
**Top 10 hits other than *Naegleria* spp.**			
Heterolobosea sp. EV10A	FM244741.1	Heterolobosea	0
Acrasis helenhemmesae BM07-A1-1	GU437220.1	Heterolobosea; Acrasida	3E-144
Tulamoeba peronaphora A1	FJ222603.1	Heterolobosea; Tulamoebidae	5E-132
Allovahlkampfia sp. SG-2014b	KF547919.1	Heterolobosea; Schizopyrenida	8E-130
Allovahlkampfia sp. SG-2014d	KF547918.1	Heterolobosea; Schizopyrenida	3E-129
Allovahlkampfia sp. SG-2014c	KF547915.1	Heterolobosea; Schizopyrenida	4E-128
Allovahlkampfia sp. SO/1P	EU266548.2	Heterolobosea; Schizopyrenida	4E-128
Allovahlkampfia spelaea SK1	EU696948.1	Heterolobosea; Schizopyrenida	4E-128
Tetramitus thermacidophilus	AJ621575.1	Heterolobosea; Schizopyrenida	1E-127
Tulamoeba sp. H3 clone 4/7	KT210085.1	Heterolobosea; Tulamoebidae	3E-127

Acanthamoeba sp. KDN1 (query; this study)	LC368141		
**Top 10 hits**			
Acanthamoeba sp. KA/E5	AY148954.1	Amoebozoa; Discosea; Longamoebia	0
Acanthamoeba sp. KA/MSS7	AY173015.1	Amoebozoa; Discosea; Longamoebia	0
Acanthamoeba sp. KA/MSS2	AY173013.1	Amoebozoa; Discosea; Longamoebia	0
Acanthamoeba sp. KA/MSS6	AY173014.1	Amoebozoa; Discosea; Longamoebia	0
Acanthamoeba sp. KA/E21	EF140633.1	Amoebozoa; Discosea; Longamoebia	0
Acanthamoeba sp. KA/E4	AF349045.1	Amoebozoa; Discosea; Longamoebia	0
Acanthamoeba sp. AcaVN14	GQ397476.1	Amoebozoa; Discosea; Longamoebia	0
Acanthamoeba griffini H37	S81337.1	Amoebozoa; Discosea; Longamoebia	0
Acanthamoeba sp. Egy4	MF350347.1	Amoebozoa; Discosea; Longamoebia	0
Acanthamoeba griffini S-7 ATCC 30731	U07412.1	Amoebozoa; Discosea; Longamoebia	0
**Top 10 hits other than *Acanthamoeba* spp.**			
Comandonia operculata	AY033896.1	Amoebozoa; Discosea; Longamoebia	0
Comandonia sp. E_5F	AB425954.1	Amoebozoa; Discosea; Longamoebia	0
Comandonia sp. E_5E	AB425956.1	Amoebozoa; Discosea; Longamoebia	0
Acanthamoebidae sp. EO-2012	JQ361081.1	Amoebozoa; Discosea; Longamoebia	0
Balamuthia mandrillaris V039	AF477019.1	Amoebozoa; Discosea; Longamoebia	0
Balamuthia mandrillaris Itson-1	KF874819.1	Amoebozoa; Discosea; Longamoebia	0
Balamuthia mandrillaris CDC:V630	JX524850.1	Amoebozoa; Discosea; Longamoebia	0
Balamuthia mandrillaris CDC:V039	AF019071.1	Amoebozoa; Discosea; Longamoebia	4E-178
Mycamoeba gemmipara	KX687875.1	Amoebozoa; Mycamoeba	7E-136
Amoebozoa sp. amMP3	JX312795.1	Amoebozoa; unclassified Amoebozoa	7E-136

Supplementary Table 1. BLAST top hits of 18S rDNA sequences of the three species of amoeba-like organisms isolated and used in this study (continued).

Species	Accession number	Classification	E-value
Vannella sp. KDN1 (query; this study)	LC368140		
**Top 10 hits**			
Vannella sp. GERL34	HM363631.1	Amoebozoa; Discosea; Flabellinia; Vannellidae	0
Vannella sp. BEN3V	JQ271727.1	Amoebozoa; Discosea; Flabellinia; Vannellidae	0
Ripella tribonemae 70.1.3	MF683568.1	Amoebozoa; Discosea; Flabellinia; Vannellidae	0
Platyamoeba sp. SUM1S/I	AY929921.1	Amoebozoa; Discosea; Flabellinia; Vannellidae	0
Vannella sp. GERL14	HM363630.1	Amoebozoa; Discosea; Flabellinia; Vannellidae	0
Vannella sp. GERB	HM363624.1	Amoebozoa; Discosea; Flabellinia; Vannellidae	
Ripella decalvata 26.2.18	MF683561.1	Amoebozoa; Discosea; Flabellinia; Vannellidae	0
Ripella platypodia 26.9.33	MF683501.1	Amoebozoa; Discosea; Flabellinia; Vannellidae	0
Ripella sp. DP13	JQ271714.1	Amoebozoa; Discosea; Flabellinia; Vannellidae	0
Vannella sp. CAZ6/I	AY929914.1	Amoebozoa; Discosea; Flabellinia; Vannellidae	0
**Top 10 hits other than Vannellidae**			
Balamuthia mandrillaris Itson-1	KF874819.1	Amoebozoa; Discosea; Longamoebia	9E-108
Amoebozoa sp. C3	KT892701.1	Amoebozoa; unclassified Amoebozoa	1E-106
Dictyamoeba vorax WalEn	KP864096.1	Amoebozoa; Dictyamoeba	2E-105
Paramoeba eilhardi CCAP1560/2	JN202438.1	Amoebozoa; Discosea; Flabellinia	6E-105
Paramoeba sp. EkV-2019b	MK168790.1	Amoebozoa; Discosea; Flabellinia	2E-104
Paramoeba branchiphila KPF3	KY465831.1	Amoebozoa; Discosea; Flabellinia	7E-104
Neoparamoeba sp. SM53	AY193726.1	Amoebozoa; Discosea; Flabellinia	7E-104
Neoparamoeba longipodia DIVA3	MF197371.1	Amoebozoa; Discosea; Flabellinia	3E-102
Neoparamoeba longipodia DIVA3 574/3	MF197370.1	Amoebozoa; Discosea; Flabellinia	3E-102
Paramoeba pemaquidensis FHL	KY465849.1	Amoebozoa; Discosea; Flabellinia	3E-102

Supplementary Table 2. Numbers of HiSeq reads that were assigned to mRNA contigs of respective species under respective culture conditions. The details of the culture conditions for RNA-seq analyses are summarized in Supplementary Fig. 3.

Naegleria sp.

Green prey	Rep. 1	Rep. 2	Rep. 3
Dark	15,406,680	15,852,082	21,460,963
Light	17,698,525	14,203,796	13,370,422

Pale prey	Rep. 1	Rep. 2	Rep. 3
Dark	14,864,745	13,907,775	12,962,365
Light	11,288,270	16,585,657	14,293,262

w/o prey	Rep. 1	Rep. 2	Rep. 3
Dark	11,393,374	15,978,300	15,696,333
Light	11,762,434	16,540,194	14,695,015
Dark+H_2O_2	12,955,437	17,445,128	14,132,034

Chl	Rep. 1	Rep. 2	Rep. 3
Dark	4,553,864	17,601,604	15,519,770
Light	6,081,370	13,064,417	13,211,099

w/o chl	Rep. 1	Rep. 2	Rep. 3
Dark	2,906,034	15,726,985	13,801,854
Light	3,081,746	16,191,173	14,001,802

RB	Rep. 1	Rep. 2	Rep. 3
Dark	8,669,676	16,586,333	13,827,326
Light	7,443,787	16,416,071	14,695,762

Acanthamoeba sp.

Green prey	Rep. 1	Rep. 2	Rep. 3
Dark	4,057,426	11,893,151	10,441,191
Light	4,679,008	12,139,051	12,116,824

Pale prey	Exp. 1
Dark	5,523,730
Light	9,998,379

w/o prey	Exp. 1
Dark	9,304,498
Light	6,421,569

Vannella sp.

Green prey	Rep. 1	Rep. 2
Dark	8,422,189	3,002,741
Light	7,394,133	4,642,215

Pale prey	Exp. 1
Dark	3,246,243
Light	8,372,368

Supplementary Table 3. GO terms enriched in upregulated genes (FDR < 0.01; edgeR; three biological replicates) when *Naegleria* sp. cultured with green *S. elongatus* prey was transferred from dark to light conditions ($p < 0.05$; GOseq)

Category	GO term		GO ID	*P*-value
-	MF	ATPase activity	GO:0016887	1.33E-06
-	MF	ATPase activity, coupled to transmembrane movement of substances	GO:0042626	9.73E-06
-	MF	zinc ion binding	GO:0008270	1.19E-05
-	MF	DNA-binding transcription factor activity, RNA polymerase II-specific	GO:0000981	1.49E-05
oxidation and reduction	BP	oxidation-reduction process	GO:0055114	1.94E-05
-	CC	Nucleus	GO:0005634	4.66E-05
-	BP	ubiquitin-dependent protein catabolic process	GO:0006511	1.20E-04
respiration	CC	Mitochondrion	GO:0005739	2.14E-04
-	BP	Transport	GO:0006810	3.19E-04
-	BP	regulation of transcription, DNA-templated	GO:0006355	3.33E-04
oxidation and reduction	MF	oxidoreductase activity	GO:0016491	3.98E-04
DNA repair	BP	DNA repair	GO:0006281	5.57E-04
-	BP	protein ubiquitination	GO:0016567	6.08E-04
-	MF	ATP binding	GO:0005524	1.03E-03
-	CC	proteasome complex	GO:0000502	1.47E-03
respiration	CC	mitochondrial inner membrane	GO:0005743	2.34E-03
-	MF	helicase activity	GO:0004386	2.67E-03
respiration	CC	mitochondrial matrix	GO:0005759	3.33E-03
oxidation and reduction	MF	oxidoreductase activity, acting on paired donors, with incorporation or reduction of molecular oxygen	GO:0016705	4.12E-03
-	BP	iron-sulfur cluster assembly	GO:0016226	4.80E-03
-	CC	chloroplast inner membrane	GO:0009706	4.83E-03
oxidative stress response	BP	response to oxidative stress	GO:0006979	5.19E-03
-	MF	heme binding	GO:0020037	5.95E-03
-	MF	ubiquitin-protein transferase activity	GO:0004842	8.41E-03
DNA repair	BP	nucleotide-excision repair	GO:0006289	8.71E-03
-	BP	protein folding	GO:0006457	9.06E-03
-	MF	serine-type endopeptidase activity	GO:0004252	0.013
-	MF	iron ion binding	GO:0005506	0.014
-	MF	aromatase activity	GO:0070330	0.015
-	BP	Proteolysis	GO:0006508	0.015
-	MF	ATP-dependent helicase activity	GO:0008026	0.016
-	MF	metal ion binding	GO:0046872	0.020
oxidation and reduction	MF	flavin adenine dinucleotide binding	GO:0050660	0.022
respiration	CC	mitochondrial intermembrane space	GO:0005758	0.024
oxidation and reduction	MF	FMN binding	GO:0010181	0.024
-	CC	integral component of membrane	GO:0016021	0.026
DNA repair	BP	base-excision repair	GO:0006284	0.029
DNA repair	MF	damaged DNA binding	GO:0003684	0.029
-	CC	melanosome	GO:0042470	0.029
-	BP	cholesterol metabolic process	GO:0008203	0.029
oxidation and reduction	MF	oxidoreductase activity, acting on paired donors, with incorporation or reduction of molecular oxygen, NAD(P)H as one donor, and incorporation of one atom of oxygen	GO:0016709	0.029
-	MF	translation initiation factor activity	GO:0003743	0.029
-	BP	response to heat	GO:0009408	0.030

Some GO terms were categorized into 6 categories: cytoskeleton and motors, DNA repair, oxidation and reduction, oxidative stress responses, phagocytosis, respiration. GO, gene ontology; MF, molecular function; CC, cellular component; and BP, biological process. The results shown are based on three independent culture sets that were performed at different days.

Supplementary Table 3. GO terms enriched in upregulated genes (FDR < 0.01; edgeR; three biological replicates) when *Naegleria* sp. cultured with green *S. elongatus* prey was transferred from dark to light conditions ($p < 0.05$; GOseq) (continued)

**Category**	**GO term**		**GO ID**	***P*-value**
DNA repair	MF	ligase activity	GO:0016874	0.033
-	BP	tRNA processing	GO:0008033	0.040
-	MF	metalloendopeptidase activity	GO:0004222	0.040
-	BP	transmembrane transport	GO:0055085	0.043
-	BP	response to drug	GO:0042493	0.044
-	CC	chloroplast envelope	GO:0009941	0.044
-	BP	mRNA processing	GO:0006397	0.044
-	MF	hydrolase activity	GO:0016787	0.049

Supplementary Table 4. GO terms enriched in downregulated genes (FDR < 0.01; edgeR; three biological replicates) when *Naegleria* sp. cultured with green *S. elongatus* prey was transferred from dark to light conditions ($p < 0.05$; GOseq)

Category	GO term		GO ID	*P*-value
cytoskeleton and motors	MF	actin filament binding	GO:0051015	4.74E-07
cytoskeleton and motors	BP	actin cytoskeleton organization	GO:0030036	5.40E-07
-	MF	calcium ion binding	GO:0005509	4.57E-06
cytoskeleton and motors	MF	actin binding	GO:0003779	5.16E-06
-	BP	cell adhesion	GO:0007155	3.05E-05
-	CC	intracellular	GO:0005622	4.87E-05
-	CC	neuron projection	GO:0043005	5.07E-05
-	MF	heparin binding	GO:0008201	6.23E-05
cytoskeleton and motors	CC	actin cytoskeleton	GO:0015629	1.36E-04
-	BP	aggregation involved in sorocarp development	GO:0031152	1.39E-04
-	BP	collagen fibril organization	GO:0030199	2.53E-04
-	CC	extracellular region	GO:0005576	2.84E-04
cytoskeleton and motors	BP	cytoskeleton organization	GO:0007010	3.11E-04
cytoskeleton and motors	CC	cytoskeleton	GO:0005856	3.44E-04
-	BP	small GTPase mediated signal transduction	GO:0007264	4.32E-04
-	BP	cell-matrix adhesion	GO:0007160	4.65E-04
-	MF	integrin binding	GO:0005178	5.06E-04
-	BP	collagen metabolic process	GO:0032963	5.06E-04
-	BP	regulation of JUN kinase activity	GO:0043506	5.06E-04
-	BP	elastic fiber assembly	GO:0048251	5.06E-04
cell motion	BP	movement of cell or subcellular component	GO:0006928	8.76E-04
cytoskeleton and motors	CC	cortical actin cytoskeleton	GO:0030864	1.57E-03
-	BP	adenylate cyclase-modulating G protein-coupled receptor signaling pathway	GO:0007188	1.57E-03
-	MF	guanyl nucleotide binding	GO:0019001	1.58E-03
-	MF	G-protein beta/gamma-subunit complex binding	GO:0031683	1.58E-03
cytoskeleton and motors	MF	motor activity	GO:0003774	1.58E-03
-	BP	triglyceride metabolic process	GO:0006641	1.69E-03
-	CC	extracellular space	GO:0005615	2.89E-03
cell motion	CC	pseudopodium	GO:0031143	3.49E-03
cell motion	CC	Filopodium	GO:0030175	3.51E-03
cytoskeleton and motors	CC	myosin complex	GO:0016459	3.54E-03
-	BP	mitotic cytokinesis	GO:0000281	3.82E-03
-	MF	protein heterodimerization activity	GO:0046982	4.21E-03
-	BP	fatty acid metabolic process	GO:0006631	4.27E-03
-	CC	cell cortex	GO:0005938	5.79E-03
-	BP	G protein-coupled receptor signaling pathway	GO:0007186	7.34E-03
cytoskeleton and motors	CC	Arp2/3 protein complex	GO:0005885	7.86E-03
-	MF	protein kinase C activity	GO:0004697	7.90E-03
-	BP	regulation of Rho protein signal transduction	GO:0035023	7.93E-03
-	BP	self proteolysis	GO:0097264	7.94E-03
-	BP	regulation of small GTPase mediated signal transduction	GO:0051056	7.94E-03
cell motion	CC	cell leading edge	GO:0031252	8.47E-03
cytoskeleton and motors	BP	actin filament polymerization	GO:0030041	8.50E-03

Some GO terms were categorized into 6 categories: cytoskeleton and motors, DNA repair, oxidation and reduction, oxidative stress responses, phagocytosis, respiration. GO, gene ontology; MF, molecular function; CC, cellular component; and BP, biological process. The results shown are based on three independent culture sets that were performed at different days.

Supplementary Table 4. GO terms enriched in downregulated genes (FDR < 0.01; edgeR; three biological replicates) when *Naegleria* sp. cultured with green *S. elongatus* prey was transferred from dark to light conditions ($p < 0.05$; GOseq) (continued)

Category	GO term		GO ID	*P*-value
cell motion	BP	chemotaxis to cAMP	GO:0043327	8.56E-03
-	BP	intracellular signal transduction	GO:0035556	9.96E-03
-	CC	Ribosome	GO:0005840	0.011
-	MF	structural constituent of ribosome	GO:0003735	0.015
-	MF	lipid binding	GO:0008289	0.016
phagocytosis	CC	phagocytic vesicle	GO:0045335	0.016
-	BP	Translation	GO:0006412	0.017
phagocytosis	BP	phagocytosis	GO:0006909	0.017
-	BP	ER to Golgi vesicle-mediated transport	GO:0006888	0.017
-	BP	cell morphogenesis	GO:0000902	0.017
-	CC	cell-cell junction	GO:0005911	0.017
-	MF	Rac GTPase binding	GO:0048365	0.017
cytoskeleton and motors	BP	Arp2/3 complex-mediated actin nucleation	GO:0034314	0.018
-	MF	phospholipase activity	GO:0004620	0.018
-	CC	extracellular exosome	GO:0070062	0.018
cell motion	BP	cell migration	GO:0016477	0.018
cytoskeleton and motors	BP	actin filament bundle assembly	GO:0051017	0.018
-	CC	intrinsic component of the cytoplasmic side of the plasma membrane	GO:0031235	0.018
-	BP	negative regulation of Ras protein signal transduction	GO:0046580	0.018
-	MF	transaminase activity	GO:0008483	0.018
-	BP	regulation of cell shape	GO:0008360	0.018
-	MF	G protein-coupled receptor binding	GO:0001664	0.018
-	CC	heterotrimeric G-protein complex	GO:0005834	0.018
-	BP	negative regulation of transcription by RNA polymerase II	GO:0000122	0.024
-	MF	signaling receptor binding	GO:0005102	0.026
-	BP	lipid catabolic process	GO:0016042	0.028
cytoskeleton and motors	BP	regulation of actin filament polymerization	GO:0030833	0.034
-	MF	Rho guanyl-nucleotide exchange factor activity	GO:0005089	0.034
phagocytosis	CC	phagocytic cup	GO:0001891	0.034
-	BP	phosphatidylinositol catabolic process	GO:0031161	0.040
-	BP	phosphatidylethanolamine catabolic process	GO:0046338	0.040
cell motion	CC	filopodium tip	GO:0032433	0.040
-	BP	cellular response to insulin stimulus	GO:0032869	0.040
cell motion	BP	Thermotaxis	GO:0043052	0.040
-	MF	hydrolase activity, acting on glycosyl bonds	GO:0016798	0.040
cell motion	BP	positive regulation of filopodium assembly	GO:0051491	0.040
-	CC	chromosome, centromeric region	GO:0000775	0.040
-	MF	calcium-dependent protein kinase C activity	GO:0004698	0.040
cytoskeleton and motors	BP	actin filament capping	GO:0051693	0.040
-	CC	cell projection	GO:0042995	0.040
-	BP	negative regulation of cell adhesion	GO:0007162	0.040
-	BP	calcium-mediated signaling	GO:0019722	0.040
cytoskeleton and motors	MF	microfilament motor activity	GO:0000146	0.040
-	BP	extracellular matrix organization	GO:0030198	0.040
cell motion	BP	Chemotaxis	GO:0006935	0.040
-	CC	integral component of plasma membrane	GO:0005887	0.040
phagocytosis	CC	phagocytic vesicle membrane	GO:0030670	0.047

Supplementary Table 5. GO terms enriched in upregulated genes (FDR < 0.01; edgeR; three biological replicates) when *Acanthamoeba* sp. cultured with green *S. elongatus* prey was transferred from dark to light conditions ($p < 0.05$; GOseq)

Category	GO term		GO ID	*P*-value
oxidation and reduction	BP	oxidation-reduction process	GO:0055114	1.66E-10
respiration	CC	Mitochondrion	GO:0005739	2.33E-10
oxidation and reduction	MF	oxidoreductase activity	GO:0016491	2.91E-06
-	BP	Proteolysis	GO:0006508	3.51E-06
-	BP	metabolic process	GO:0008152	4.39E-06
oxidation and reduction	MF	flavin adenine dinucleotide binding	GO:0050660	6.01E-06
DNA repair	BP	DNA repair	GO:0006281	6.44E-05
-	MF	ATPase activity	GO:0016887	6.90E-05
-	MF	metal ion binding	GO:0046872	1.03E-04
-	CC	proteasome complex	GO:0000502	1.09E-04
respiration	CC	mitochondrial inner membrane	GO:0005743	1.47E-04
-	BP	proteolysis involved in cellular protein catabolic process	GO:0051603	1.84E-04
-	CC	integral component of membrane	GO:0016021	2.43E-04
-	MF	threonine-type endopeptidase activity	GO:0004298	3.15E-04
-	CC	proteasome core complex	GO:0005839	3.15E-04
DNA repair	MF	ligase activity	GO:0016874	3.20E-04
respiration	CC	mitochondrial matrix	GO:0005759	3.32E-04
-	MF	pyridoxal phosphate binding	GO:0030170	9.45E-04
-	CC	Nucleus	GO:0005634	1.12E-03
-	BP	biosynthetic process	GO:0009058	1.63E-03
-	BP	ubiquitin-dependent protein catabolic process	GO:0006511	2.32E-03
-	BP	protein catabolic process	GO:0030163	2.75E-03
oxidation and reduction	MF	oxidoreductase activity, acting on the CH-CH group of donors	GO:0016627	2.78E-03
-	BP	Transport	GO:0006810	4.29E-03
-	BP	sorocarp morphogenesis	GO:0031288	4.76E-03
-	MF	iron ion binding	GO:0005506	7.73E-03
oxidation and reduction	MF	iron-sulfur cluster binding	GO:0051536	8.02E-03
oxidation and reduction	MF	4 iron, 4 sulfur cluster binding	GO:0051539	8.08E-03
-	MF	acyl-CoA dehydrogenase activity	GO:0003995	8.17E-03
-	MF	endopeptidase activity	GO:0004175	0.014
-	BP	DNA replication	GO:0006260	0.014
-	BP	glycolytic process	GO:0006096	0.014
-	MF	metalloendopeptidase activity	GO:0004222	0.014
-	CC	proteasome accessory complex	GO:0022624	0.014
oxidation and reduction	MF	2 iron, 2 sulfur cluster binding	GO:0051537	0.014
-	MF	ubiquitin-protein transferase activity	GO:0004842	0.015
-	CC	Peroxisome	GO:0005777	0.015
-	MF	hydrolase activity	GO:0016787	0.017
-	CC	endoplasmic reticulum membrane	GO:0005789	0.017
-	BP	transmembrane transport	GO:0055085	0.022
oxidation and reduction	MF	electron transfer activity	GO:0009055	0.023
-	CC	proteasome core complex, alpha-subunit complex	GO:0019773	0.024
oxidation and reduction	BP	fatty acid beta-oxidation	GO:0006635	0.024
-	BP	steroid biosynthetic process	GO:0006694	0.024
-	BP	protein folding	GO:0006457	0.024
-	MF	ATPase activity, coupled to transmembrane movement of substances	GO:0042626	0.025
-	MF	catalytic activity	GO:0003824	0.030

Some GO terms were categorized into 6 categories: cytoskeleton and motors, DNA repair, oxidation and reduction, oxidative stress responses, phagocytosis, respiration. GO, gene ontology; MF, molecular function; CC, cellular component; and BP, biological process. The results shown are based on three independent culture sets that were performed at different days.

Supplementary Table 5. GO terms enriched in upregulated genes (FDR < 0.01; edgeR; three biological replicates) when *Acanthamoeba* sp. cultured with green *S. elongatus* prey was transferred from dark to light conditions ($p < 0.05$; GOseq) (continued)

**Category**	**GO term**		**GO ID**	***P*-value**
-	BP	protein transport	GO:0015031	0.038
-	BP	cell differentiation	GO:0030154	0.038
respiration	MF	NADH dehydrogenase (ubiquinone) activity	GO:0008137	0.040
oxidation and reduction	MF	oxidoreductase activity, acting on the CH-OH group of donors, NAD or NADP as acceptor	GO:0016616	0.041
-	BP	cholesterol biosynthetic process	GO:0006695	0.041
-	MF	metallopeptidase activity	GO:0008237	0.041

Supplementary Table 6. GO terms enriched in downregulated genes (FDR < 0.01; edgeR; three biological replicates) when *Acanthamoeba* sp. cultured with green *S. elongatus* prey was transferred from dark to light conditions ($p < 0.05$; GOseq)

Category	GO term		GO ID	*P*-value
-	MF	protein serine/threonine kinase activity	GO:0004674	4.10E-09
-	MF	Rho guanyl-nucleotide exchange factor activity	GO:0005089	4.24E-08
-	BP	intracellular signal transduction	GO:0035556	6.39E-08
cytoskeleton and motors	CC	myosin complex	GO:0016459	1.04E-07
-	BP	regulation of Rho protein signal transduction	GO:0035023	2.35E-07
cytoskeleton and motors	MF	motor activity	GO:0003774	2.56E-07
-	CC	cell cortex	GO:0005938	3.03E-07
-	BP	signal transduction	GO:0007165	5.09E-07
-	BP	regulation of small GTPase mediated signal transduction	GO:0051056	1.29E-06
-	MF	GTPase activator activity	GO:0005096	1.59E-06
-	BP	protein phosphorylation	GO:0006468	2.47E-06
cytoskeleton and motors	CC	cytoskeleton	GO:0005856	2.88E-06
-	BP	small GTPase mediated signal transduction	GO:0007264	8.51E-06
-	MF	protein kinase activity	GO:0004672	8.69E-06
-	BP	cyclic nucleotide biosynthetic process	GO:0009190	1.01E-05
-	MF	phosphorus-oxygen lyase activity	GO:0016849	1.01E-05
cell motion	CC	cell leading edge	GO:0031252	2.27E-05
cytoskeleton and motors	MF	actin filament binding	GO:0051015	3.66E-05
cytoskeleton and motors	MF	actin binding	GO:0003779	5.68E-05
-	CC	intracellular	GO:0005622	7.13E-05
-	MF	Rac GTPase binding	GO:0048365	1.42E-04
-	BP	mitotic cytokinesis	GO:0000281	3.12E-04
-	MF	guanyl-nucleotide exchange factor activity	GO:0005085	3.14E-04
cell motion	CC	pseudopodium	GO:0031143	3.29E-04
cell motion	CC	lamellipodium	GO:0030027	3.36E-04
phagocytosis	CC	early phagosome	GO:0032009	3.42E-04
cell motion	CC	filopodium	GO:0030175	8.09E-04
cell motion	BP	chemotaxis	GO:0006935	8.19E-04
phagocytosis	CC	phagocytic cup	GO:0001891	8.23E-04
-	NA	NA	GO:0032320	8.40E-04
-	MF	calcium ion binding	GO:0005509	8.96E-04
-	BP	protein dephosphorylation	GO:0006470	9.16E-04
cytoskeleton and motors	CC	cortical actin cytoskeleton	GO:0030864	1.97E-03
cytoskeleton and motors	BP	regulation of actin filament polymerization	GO:0030833	1.99E-03
-	MF	protein binding	GO:0005515	3.57E-03
-	BP	Ras protein signal transduction	GO:0007265	4.73E-03
-	BP	cell morphogenesis	GO:0000902	4.87E-03
cell motion	CC	ruffle	GO:0001726	4.91E-03
phagocytosis	BP	phagocytosis	GO:0006909	5.04E-03
actomyosin	BP	actin cytoskeleton organization	GO:0030036	5.12E-03
-	BP	sorocarp development	GO:0030587	5.18E-03
phagocytosis	CC	phagocytic vesicle membrane	GO:0030670	9.96E-03
-	BP	cell-substrate adhesion	GO:0031589	0.012
cytoskeleton and motors	BP	actin filament-based movement	GO:0030048	0.012
-	MF	protein serine/threonine phosphatase activity	GO:0004722	0.012
-	BP	exocytosis	GO:0006887	0.015

Some GO terms were categorized into 6 categories: cytoskeleton and motors, DNA repair, oxidation and reduction, oxidative stress responses, phagocytosis, respiration. GO, gene ontology; MF, molecular function; CC, cellular component; and BP, biological process. The results shown are based on three independent culture sets that were performed at different days.

Supplementary Table 6. GO terms enriched in downregulated genes (FDR < 0.01; edgeR; three biological replicates) when *Acanthamoeba* sp. cultured with green *S. elongatus* prey was transferred from dark to light conditions ($p < 0.05$; GOseq) (continued)

Category	GO term		GO ID	*P*-value
-	MF	phosphoprotein phosphatase activity	GO:0004721	0.015
-	NA	NA	GO:0005099	0.015
-	CC	intrinsic component of the cytoplasmic side of the plasma membrane	GO:0031235	0.015
-	MF	protein tyrosine phosphatase activity	GO:0004725	0.019
-	NA	NA	GO:0032318	0.022
cytoskeleton and motors	CC	Actomyosin	GO:0042641	0.022
cell motion	CC	cell projection	GO:0042995	0.022
-	BP	aggregation involved in sorocarp development	GO:0031152	0.026
phagocytosis	CC	phagocytic cup lip	GO:0097203	0.029
cytoskeleton and motors	CC	Arp2/3 protein complex	GO:0005885	0.029
cytoskeleton and motors	BP	Arp2/3 complex-mediated actin nucleation	GO:0034314	0.029
cytoskeleton and motors	BP	actin filament polymerization	GO:0030041	0.029
-	BP	positive regulation of protein kinase B signaling	GO:0051897	0.029
cytoskeleton and motors	MF	actin-dependent ATPase activity	GO:0030898	0.029
cell motion	BP	filopodium assembly	GO:0046847	0.029
-	BP	establishment or maintenance of cell polarity	GO:0007163	0.029
-	BP	cell-cell junction organization	GO:0045216	0.029
cytoskeleton and motors	BP	actin filament bundle assembly	GO:0051017	0.029
cell motion	BP	axon guidance	GO:0007411	0.029
-	MF	phosphatidylinositol-3-phosphatase activity	GO:0004438	0.029
-	MF	Ras guanyl-nucleotide exchange factor activity	GO:0005088	0.029
-	BP	regulation of cell shape	GO:0008360	0.029
-	MF	Rac guanyl-nucleotide exchange factor activity	GO:0030676	0.029
-	BP	negative regulation of Ras protein signal transduction	GO:0046580	0.030
cytoskeleton and motors	BP	actin filament organization	GO:0007015	0.047
cytoskeleton and motors	BP	actin crosslink formation	GO:0051764	0.047
-	BP	phosphatidylinositol dephosphorylation	GO:0046856	0.047
-	CC	Centrosome	GO:0005813	0.047

Supplementary Table 7. Primers used for quantitative RT-PCR in this study (Fig. 4b and Supplementary Fig. 6)

Primer name	Sequence (5'-3')
EF1-alpha-F	AACGTTGTCGTCATTGGTCA
EF1-alpha-R	CTTGTCCAAAACCCAAGCAT
Phytoene desaturase-F	GATGCTGGAAAGGATTCTGC
Phytoene desaturase-R	TCAACCCATTCCTCGTAACC
DNA-(apurinic or apyrimidinic site) lyase-F	ATCGATTGGAGTGGGTGAAA
DNA-(apurinic or apyrimidinic site) lyase-R	GGGGATGGCTTTGAAGATTT
Deoxyribodipyrimidine photo-lyase-F	TGGGAAATACCAACAGACCA
Deoxyribodipyrimidine photo-lyase-R	CAGCACCCATTCTCCAATCT
Myosin type I-F	CAGCCAGCTCTCAACCAAAG
Myosin type I-R	TCGTCATCTGCTGATGCTTG
Myosin type II-F	AGCTGGTCTCGTTCGTGAAA
Myosin type II-R	GCCTTGACGAATGGAATTGA
Myosin family-F	CACAAGCTGCATGTCAAGGT
Myosin family-R	AACGATCAGCCTCAACCAAT
Actin-F	GCCGAAAGAGAAATCGTCAG
Actin-R	CCAGCAGCTTCCATACCAAT
COX15-F	TGCTGCAAAGGGATTCTTCT
COX15-R	GCCAAATCGAAACTCTTCCA
Glutathione peroxidase (comp227059)-F	CATCAGCATGAGCGAAATGA
Glutathione peroxidase (comp227059)-R	GGTCTTTGGTGAAGGCACAA
Glutathione peroxidase (comp226183)-F	TGATGCACAAGGAAACGATG
Glutathione peroxidase (comp226183)-R	TATCTCTGGCGAATGTGCAA
PAO-like 1-F	GATTCCAACTGGTCAAAACGA
PAO-like 1-R	CCCTTAATTGGAGCATCAGC
PAO-like 2-F	TCCAAACGAAACCTCATTCC
PAO-like 2-R	ACGAAACATGGAGGGACAAA
