# Supplementary material for: Responses of unicellular predators to cope with the phototoxicity of photosynthetic prey
Source: Nat Commun. 2019 Dec 6;10:5606. doi: 10.1038/s41467-019-13568-6 (PMC6898599; doi:10.1038/s41467-019-13568-6)
Supplement: Supplementary file 3 — Reporting Summary [file 41467_2019_13568_MOESM3_ESM.pdf]

nature research

Corresponding author(s): Shin-ya Miyagishima

Last updated by author(s): Oct 28, 2019

Reporting Summary

Nature Research wishes to improve the reproducibility of the work that we publish. This form provides structure for consistency and transparency in reporting. For further information on Nature Research policies, see Authors & Referees and the Editorial Policy Checklist .

Statistics

For all statistical analyses, confirm that the following items are present in the figure legend, table legend, main text, or Methods section.

n/a	Confirmed	
☐	☒	The exact sample size (n) for each experimental group/condition, given as a discrete number and unit of measurement
☐	☒	A statement on whether measurements were taken from distinct samples or whether the same sample was measured repeatedly
☐	☒	The statistical test(s) used AND whether they are one- or two-sided<br>*Only common tests should be described solely by name; describe more complex techniques in the Methods section.*
☒	☐	A description of all covariates tested
☒	☐	A description of any assumptions or corrections, such as tests of normality and adjustment for multiple comparisons
☐	☒	A full description of the statistical parameters including central tendency (e.g. means) or other basic estimates (e.g. regression coefficient) AND variation (e.g. standard deviation) or associated estimates of uncertainty (e.g. confidence intervals)
☐	☒	For null hypothesis testing, the test statistic (e.g. F, t, r) with confidence intervals, effect sizes, degrees of freedom and P value noted<br>*Give P values as exact values whenever suitable.*
☐	☒	For Bayesian analysis, information on the choice of priors and Markov chain Monte Carlo settings
☒	☐	For hierarchical and complex designs, identification of the appropriate level for tests and full reporting of outcomes
☒	☐	Estimates of effect sizes (e.g. Cohen's d, Pearson's r), indicating how they were calculated

Our web collection on statistics for biologists contains articles on many of the points above.

Software and code

Policy information about availability of computer code

Data collection	Regarding microscopic images, fluorescent images (and corresponding light-filed images) were obtained by Metamorph (Molecular Deveces). Other images (not for further quantitative analyses) were obtained by DP controller (Olympus) (fluorescence and/or blight filed). RNA-seq data were obtained as described in the manuscript.
Data analysis	Software used to analyze microscopic images and RNA-seq data and for phylogenetic analyses are described in the manuscript.

For manuscripts utilizing custom algorithms or software that are central to the research but not yet described in published literature, software must be made available to editors/reviewers. We strongly encourage code deposition in a community repository (e.g. GitHub). See the Nature Research guidelines for submitting code & software for further information.

Data

Policy information about availability of data

All manuscripts must include a data availability statement. This statement should provide the following information, where applicable:

- Accession codes, unique identifiers, or web links for publicly available datasets
- A list of figures that have associated raw data
- A description of any restrictions on data availability

All accession codes for RNA-seq raw data are described in the manuscript.

Field-specific reporting

Please select the one below that is the best fit for your research. If you are not sure, read the appropriate sections before making your selection.

Life sciences study design

All studies must disclose on these points even when the disclosure is negative.

Sample size	The size of culture (volume of the medium and cell number/optical density) for RNA extraction and the amount of total RNA used for RNA-seq analyses are described in the manuscript. Regarding the quantitative analyses of microscopic images, ≥100 cells were analyzed to obtain a single data point.
Data exclusions	No data were excluded from the analyses.
Replication	Number of replicates for respective analyses are described in the manuscript.
Randomization	This was not relevant to our study.
Blinding	This was not relevant to our study.

Reporting for specific materials, systems and methods

We require information from authors about some types of materials, experimental systems and methods used in many studies. Here, indicate whether each material, system or method listed is relevant to your study. If you are not sure if a list item applies to your research, read the appropriate section before selecting a response.

Materials & experimental systems

n/a	Involved in the study	
☒	☐	Antibodies
☐	☒	Eukaryotic cell lines
☒	☐	Palaeontology
☒	☐	Animals and other organisms
☒	☐	Human research participants
☒	☐	Clinical data

Methods

n/a	Involved in the study	
☒	☐	ChIP-seq
☒	☐	Flow cytometry
☒	☐	MRI-based neuroimaging

Eukaryotic cell lines

Policy information about cell lines

Cell line source(s)	Cell lines were newly established in this study from environmental samples as described in the manuscript.
Authentication	We will share the cell lines upon requests.
Mycoplasma contamination	All cell lines do not contain any mycoplasma contamination.
Commonly misidentified lines (See ICLAC register)	None
