# Supplementary material for: Responses of unicellular predators to cope with the phototoxicity of photosynthetic prey
Source: Nat Commun. 2019 Dec 6;10:5606. doi: 10.1038/s41467-019-13568-6 (PMC6898599; doi:10.1038/s41467-019-13568-6)
Supplement: Supplementary file 4 — Description of Additional Supplementary Files [file 41467_2019_13568_MOESM4_ESM.pdf]

Description of Additional Supplementary Files

File Name: Supplementary Data 1
Description: FPKM values of mRNA contigs under respective cultivation conditions in Naegleria sp., Acanthamoeba sp., and Vannella sp.

File Name: Supplementary Data 2
Description: KEGG classification of up- and downregulated contigs (FDR < 0.01; edgeR; three biological replicates) when Naegleria sp. and Acanthamoeba sp. cultured with green S. elongatus prey were transferred from dark to light conditions.
